# Recent advances in chiral nanomaterials with unique electric and magnetic properties

**DOI:** 10.1186/s40580-022-00322-w

**Published:** 2022-07-18

**Authors:** Junyoung Kwon, Won Jin Choi, Uichang Jeong, Wookjin Jung, Inkook Hwang, Ki Hyun Park, Seowoo Genevieve Ko, Sung Min Park, Nicholas A. Kotov, Jihyeon Yeom

**Affiliations:** 1 Department of Materials Science and Engineering, Korea Advanced Institute of Science and Technology (KAIST), Daejeon, 34141 Republic of Korea; 2 Department of Materials Science and Engineering, University of Michigan, Ann Arbor, MI 48109 USA; 3 Lawrence Livermore National Laboratory, 7000 East Ave., Livermore, CA 94551 USA; 4 Department of Chemical Engineering, University of Michigan, Ann Arbor, MI 48109 USA; 5 Biointerfaces Institute, University of Michigan, Ann Arbor, MI 48109 USA; 6 Department of Biological Sciences, Korea Advanced Institute of Science and Technology (KAIST), Daejeon, 34141 Republic of Korea; 7 Institute for Health Science and Technology, Korea Advanced Institute of Science and Technology (KAIST), Daejeon, 34141 Republic of Korea; 8 Institute for the Nanocentury, Korea Advanced Institute of Science and Technology (KAIST), Daejeon, 34141 Republic of Korea

**Keywords:** Chiral nanomaterials, Inorganic nanomaterials, Organic–inorganic hybrid nanomaterials, Electromagnetic properties, Biomimetic nanostructures

## Abstract

Research on chiral nanomaterials (NMs) has grown radically with a rapid increase in the number of publications over the past decade. It has attracted a large number of scientists in various fields predominantly because of the emergence of unprecedented electric, optical, and magnetic properties when chirality arises in NMs. For applications, it is particularly informative and fascinating to investigate how chiral NMs interact with electromagnetic waves and magnetic fields, depending on their intrinsic composition properties, atomic distortions, and assembled structures. This review provides an overview of recent advances in chiral NMs, such as semiconducting, metallic, and magnetic nanostructures.

## Introduction

Chirality describes the geometrical property of an object when it is not superimposable with its mirror image like our left and right hands, which is extendable to nanoscale objects such as geometrical objects built from nanoparticles [1]. It is ubiquitous in nature and numerous chiral substances are present in our body exemplified by amino acids that exist only as left-handed, l-enantiomers, and sugars exist as right-handed, d-enantiomers [2]. Homochirality of these molecules is essential for biological structures forming complex living systems [3] and is considered to be a physiological process showing high stereo-selectivity, which endows chiral with a biological system, depending on their handedness [4]. Along with biochemistry, chirality is also common in physics. For example, the spin of an electron is makes it chiral [5], and electromagnetic waves propagate in a left-handed or a right-handed form [6], implying that the handedness of chiral substances can be measured using an optical instrument using a left-handed (LCP) or right-handed circularly polarized light (RCP) [7].

**Fig. 1:**
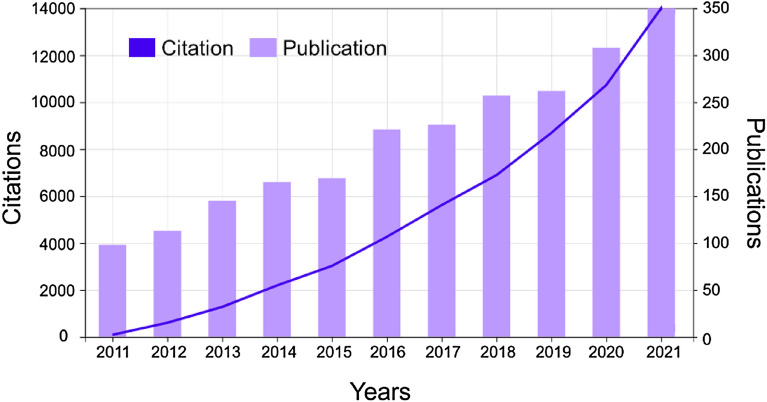
The number of annual citations and publications from 2011 to 2021. Searches were carried out with Web of Science using the keyword: chiral nanomaterials

Motivated by the chirality in organic chemistry, the wave optics and the interesting properties of NMs, materials scientists have extensively studied chiral NMs over the past decade (Fig. 1). They have found that new physicochemical properties have arisen when NMs become chiral [8–11]. For example, the assembly of several gold nanoparticles gave rise to the new chiroptical peaks in the near-infrared part of the optical spectrum and the magnetic modulation of optical properties has been realized by introducing chiral distortions in the achiral inorganic core by the chiral organic ligands [9]. In addition, chiral building blocks can be hierarchically self-organized with high complexity, much higher than those emerging from their biological counterparts in terms of graph theory [12]. Therefore, it is intriguing to investigate the various electromagnetic properties of chiral NMs, their mutual interactions, and their possible impacts on biological systems. It is clear that the chirality of NMs can be derived from different origins depending on the synthesis method, which can be categorized into three main types. (1) The crystal lattice of the inorganic part of NMs can display chirality due to atomic or interplanar distortion induced by chiral ligands (e.g., chiral ceramic NMs [13] and chiral perovskites [14]) or intrinsic symmetry of its crystal lattice. (2) The inorganic core can be chemically ‘sculpted’ to have chiral shapes via biased crystallization or self-assembly (e.g., propeller-like gold nanostructures, self-assembled gold nanorod helices [15].) (3) Light-matter interactions with photons can result in mirror transient or permanent asymmetry exemplified by the chiral electronic state of inorganic part of NMs (e.g., gold nanorods protected with a chiral thiol monolayer [16]). The chiral NMs which will be discussed in this review have been generally synthesized or assembled through bottom-up strategies.

**Fig. 2:**
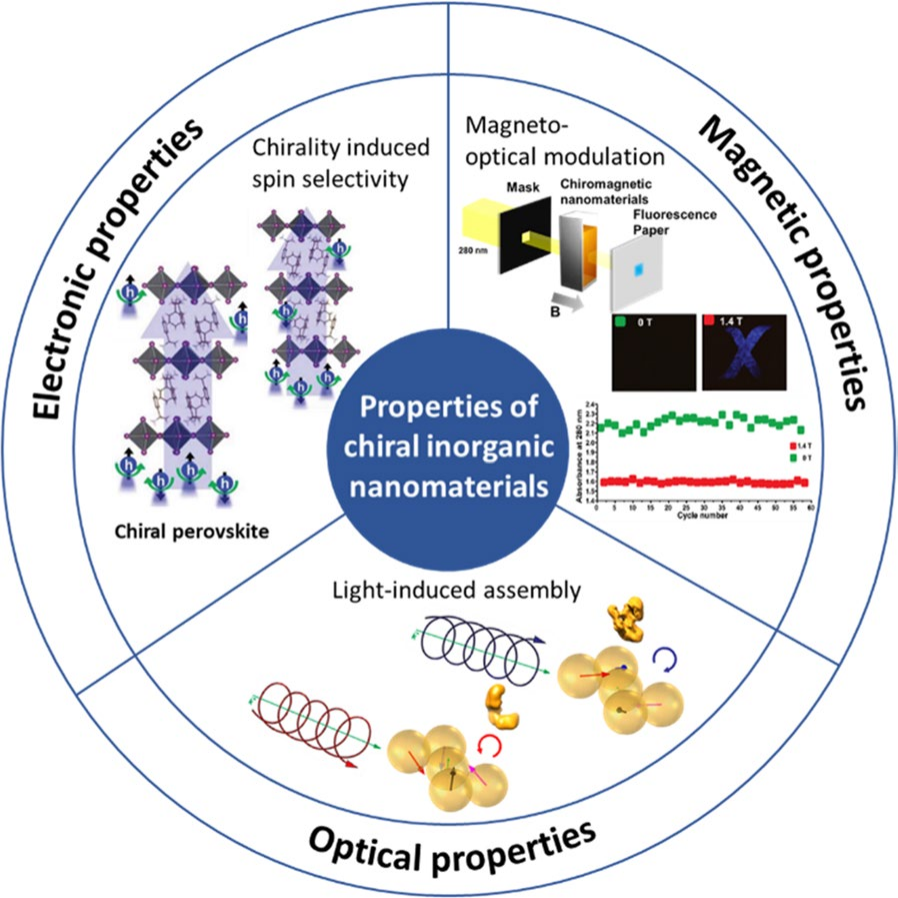
Representative chiral NMs with different properties composed with pictures from [9, 14, 17, 18]

This review covers a brief introduction of synthesis methods of emerging chiral NMs and their electronic, magnetic, and electromagnetic properties (Fig. 2). In particular, Sect. 2 describes the electronic interactions of chiral NMs. Section 3 presents chiromagnetic NMs and their magnetic contributions to the chiroptical activity of NMs. Finally, in Sect. 4, we introduce optical interactions of chiral NMs, including light-induced chiral self-assembly of semiconducting and metallic NMs.

## Electronic properties of chiral NMs

### Chirality induced spin selectivity (CISS) effect

The energy level of electrons with opposite spin states can be split when the magnetic field is applied. Such splitting can favor some reactions by providing a reaction path via a spin-specific lower energy state, which are attractive directions for catalysis, optoelectronics, and spintronics. However, engineering of NMs with preferred spin has challenges emerging primarily from the little difference in energy between the split levels that greatly limit practical application and necessity of high magnetic field. Thus, a new approach to solving these problems is urgently needed.

**Fig. 3:**
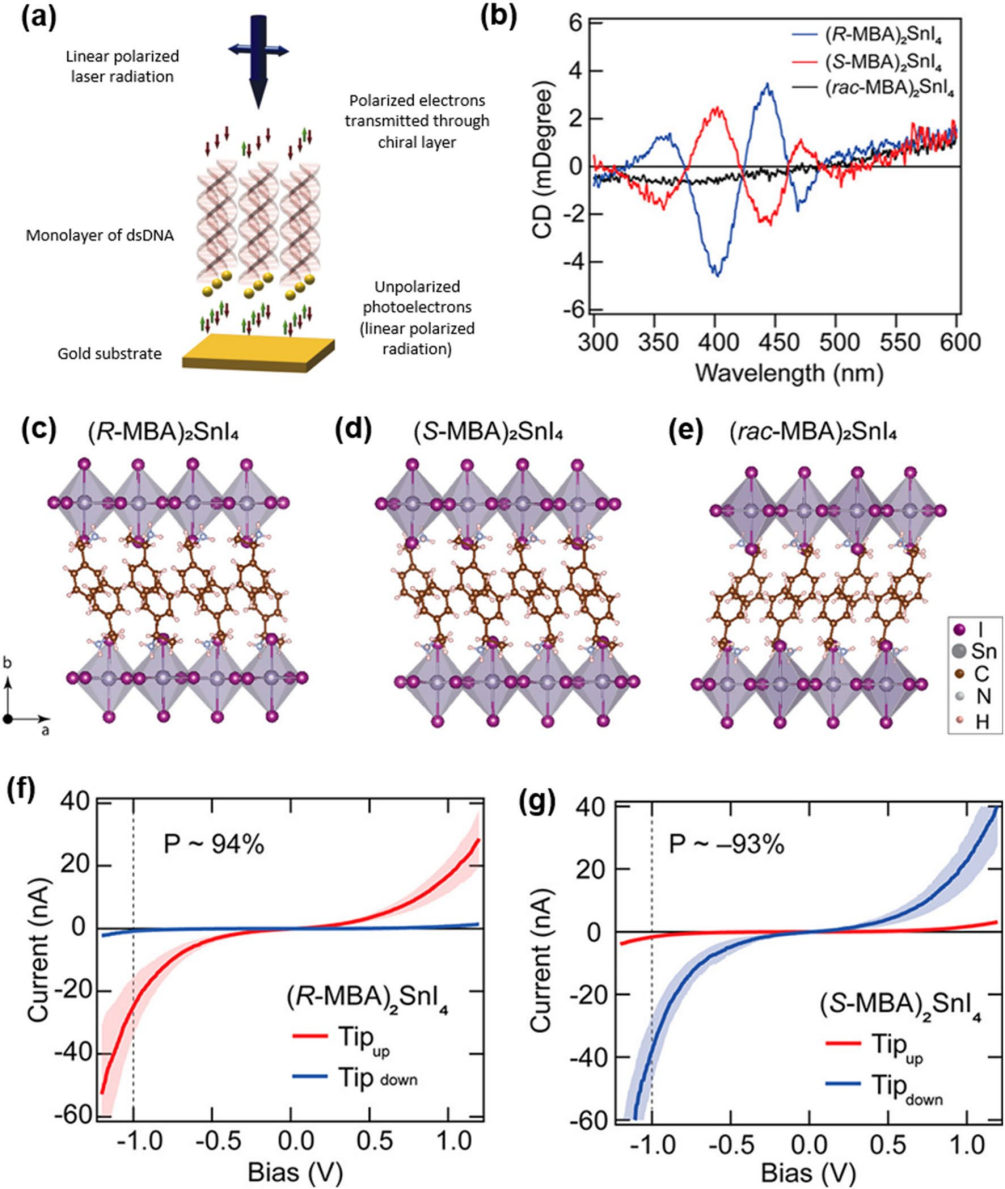
**a** Schematics of the monolayer of dsDNA on a gold substrate as a spin filter. Reproduced with permission from [23]. Copyright 2011 AAAS. **b** CD signals of chiral and racemic HOIPs. Crystal structure of chiral HOIPs with **c** *R*-MBA, **d** *S*-MBA, and **e** *rac*-MBA. Room-temperature *J*–*V* curves obtained using the magnetic conductive-probe atomic force microscopy (AFM) technique of chiral 2D hybrid (*R*-MBA)_2_SnI_4_ **f** and (*S*-MBA)_2_SnI_4_ **g** thin films. Reproduced with permission from [14]. Copyright American Chemical Society, 2020

The charge polarization in chiral molecules at interfaces creates a magnetic field owing to the broken mirror-image symmetry. The broken image symmetry induces a preferred spin state based on chiral handedness because the induced magnetic field interacts with the spin of electrons moving in chiral molecules. This unique property was first described in 1999 and was named chiral-induced spin selectivity (CISS), by Ron Naaman in 2006 [19, 20],

$$\overrightarrow{B}=\frac{\overrightarrow{v}}{{c}^{2}} \times {\overrightarrow{E}}_{chiral}$$

where $\overrightarrow{B}$ refers to a generated magnetic field, $\overrightarrow{v}$ is the velocity of the electron, c is the speed of light, and ${\overrightarrow{E}}_{chiral}$ is the electric field occurred by moving electrons in chiral molecules [21]. The induced magnetic field breaks spin degeneracy in the same orbital due to spin–orbit coupling (SOC). Notably, a previous report demonstrated that both SOC and chirality are essential for inducing spin polarization [22]. For instance, chiral double-stranded DNA monolayers on gold substrates induced the CISS effect. Spin polarizations were measured quantitatively at room temperature in 2011 (Fig. 3a), implying that the CISS effect can be used in spintronic devices to induce preferred spin states [23]. Carbon nanotubes with large SOC [24, 25] exhibited the CISS effect. The calculated impact of CISS in carbon nanotubes indicates that a high SOC value can lead to high spin polarization [26, 27]. Various chiral materials such as oligopeptides, chiral ligand-capped CdSe quantum dots, and metal organic frameworks also have been used in spin devices to achieve the CISS [28–30] and these results demonstrated that the CISS effect can be a key to achieving high spin selectivity without magnetic field.

### Chiral hybrid organic–inorganic perovskites

#### 2D chiral perovskites

Recently, hybrid organic–inorganic perovskites (HOIPs) have been utilized for CISS-based spin devices because of their large SOC abilities and tunable Rashba splitting [31, 32]. Chiral HOIPs also provide unique properties such as solution processability, tunability, and long electron-spin relaxation times, which benefit optoelectronic devices [33].

The chiral structure of HOIPs was achieved by introducing a chiral organic cation to the A site of the perovskite. Especially in 2D layered HOIPs, the chiral organic cations should have benzene rings to induce strong lattice distortion. The chirality of the methylbenzylammonium (MBA) cation is successfully transferred to the inorganic sublattices, and circular dichroism (CD) signals of chiral HOIPs exhibit mirror-symmetric CD values [34]. The structural change in perovskite is mainly due to the π–π interaction of the benzene ring in the MBA cation. The 2D inorganic layers are connected by the MBA cation, and the benzene ring of the adjacent MBA cation interacts with each other via π–π interactions (Fig. 3c–e). Chirality of the MBA cation also induces broken symmetry of inorganic layers through the hydrogen bonds. The structural parameters confirm the structural distortion of the HOIPs. The electronic and optical properties of the distorted inorganic layers are further elucidated by carrying out experimental measurements and DFT-based calculations. The chiral MBA cation transfers its chirality to the excitonic transition states and charge transfer states of chiral HOIPs (Fig. 3b), which enable the 2D-layered chiral HOIP film to work as a spin filter with a spin polarization of up to 94% by the CISS effect, demonstrating the potential use for the future spin device applications [14].

**Fig. 4:**
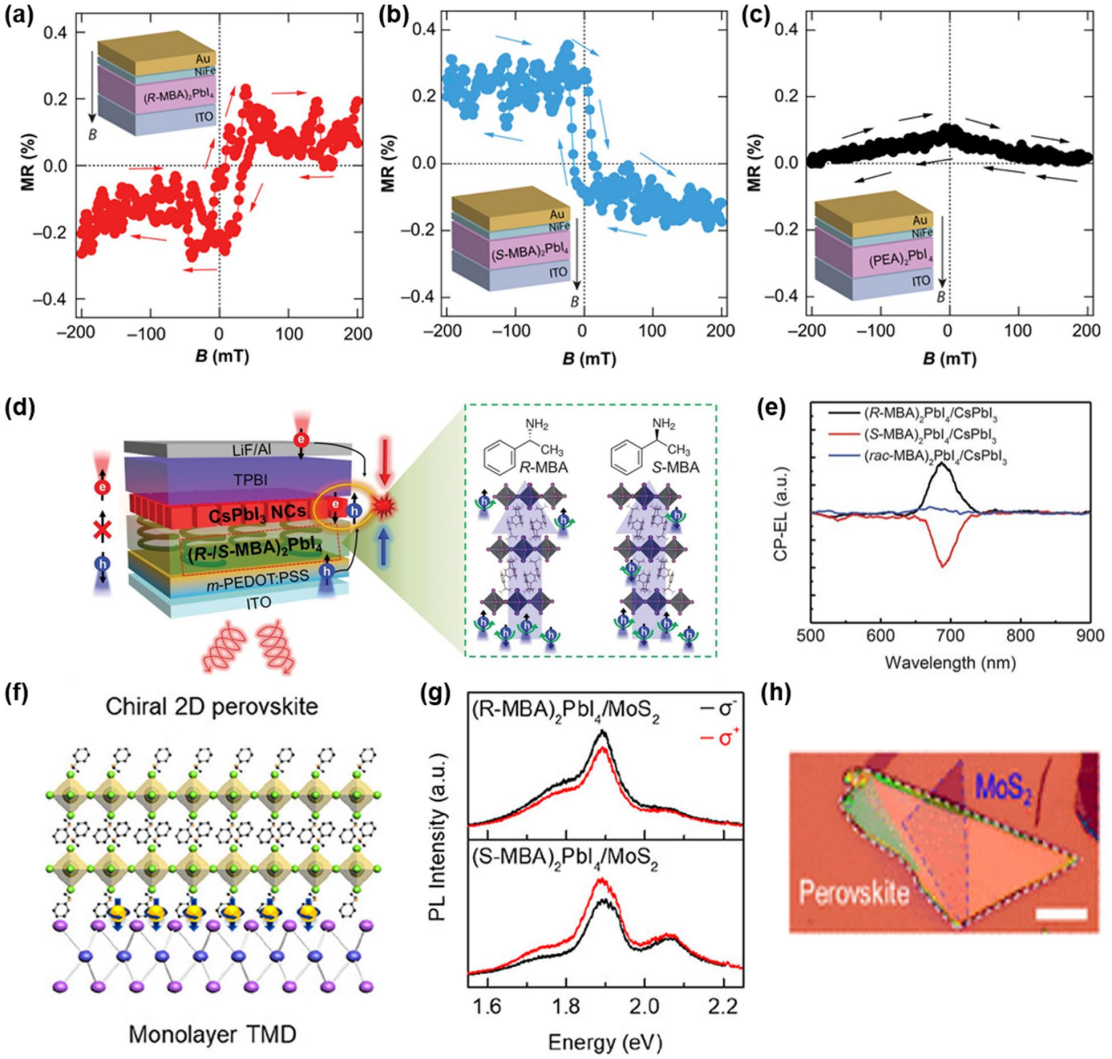
Magnetoresistance response of spintronic devices. **a** (*S*-MBA)_2_PbI_4,_ **b** (*R*-MBA)_2_PbI_4_, and **c** nonchiral HOIPs. Reproduced with permission from [37]. Copyright 2019 AAAS. **d** Schematic description of CP-EL emission by spin polarization in the spin-LEDs. **e** The CP-EL spectra of chiral and nonchiral HOIPs based spin LEDs. Reproduced with permission from [35]. Copyright 2021 AAAS. **f** Schematic structure of chiral 2D HOIPs/TMD heterostructures. **g** Photoluminescence spectra of chiral 2D HOIPs/TMD heterostructures. **h** Optical image of chiral 2D HOIPs/TMD heterostructures. Reproduced with permission from [36]. Copyright American Chemical Society, 2020

Instead of traditional spin-valve devices with two ferromagnetic electrodes, chiral HOIP spin-valve devices consisting of a single chiral HOIP spin filter layer and a single ferromagnetic electrode were fabricated. The magnetoresistance responses of the chiral HOIP spintronic devices are contradictory because of the opposite spin polarization induced by the chirality of perovskite layers (Fig. 4a, b). These magnetoresistance responses of (*R*-MBA)_2_PbI_4_-, (*S*-MBA)_2_PbI_4_, and achiral HOIP-based spin devices demonstrate that the CISS effect of chiral HOIPs successfully induces spin polarization and the chiral HOIPs can be used for spintronic devices.

The CISS of chiral HOIPs enabled spin state control and made spin-polarized light-emitting diodes (spin-LEDs) workable at room temperature without magnetic fields nor ferromagnetic layers [35]. The chiral 2D layered (*R*/*S*-MBA)_2_PbI_4_ films induce spin-polarized hole current, and the polarized carriers recombine with electrons in the emitting layer where circularly polarized electroluminescence (CP-EL) is emitted (Fig. 4d). The spin-LEDs have opposite CP-EL with opposite chirality of HOIPs and exhibit an external quantum efficiency of over 10% (Fig. 4e). Finally, the device achieved ± 2.6% CP-EL at room temperature without an external magnetic field, demonstrating that the chiral HOIPs can be promising candidates for future CP-EL devices.

By introducing chiral HOIPs to monolayer 2D transition metal dichalcogenide (TMD)-based valleytronic devices, selective spin injection to the monolayer TMDs was induced using chiral HOIPs without an external magnetic field, achieving valley polarization of over 10% under linearly polarized excitation (Fig. 4f–h) [36]. This result provides a new approach to controlling valley polarization in TMDs and demonstrates the versatility of chiral 2D HOIPs for various optoelectronic devices.

#### 1D chiral perovskites

As seen in the previous section, 2D perovskites have been successfully synthesized and analyzed for applications to various electronic devices. These results show the versatility of 2D chiral HOIPs. However, insufficient CD values and low carrier mobility due to the huge size of chiral cations have limited further applications. As alternatives, 1D chiral HOIPs have been suggested as promising candidates for future spintronic devices for high CD values and *g*-factors.

**Fig. 5:**
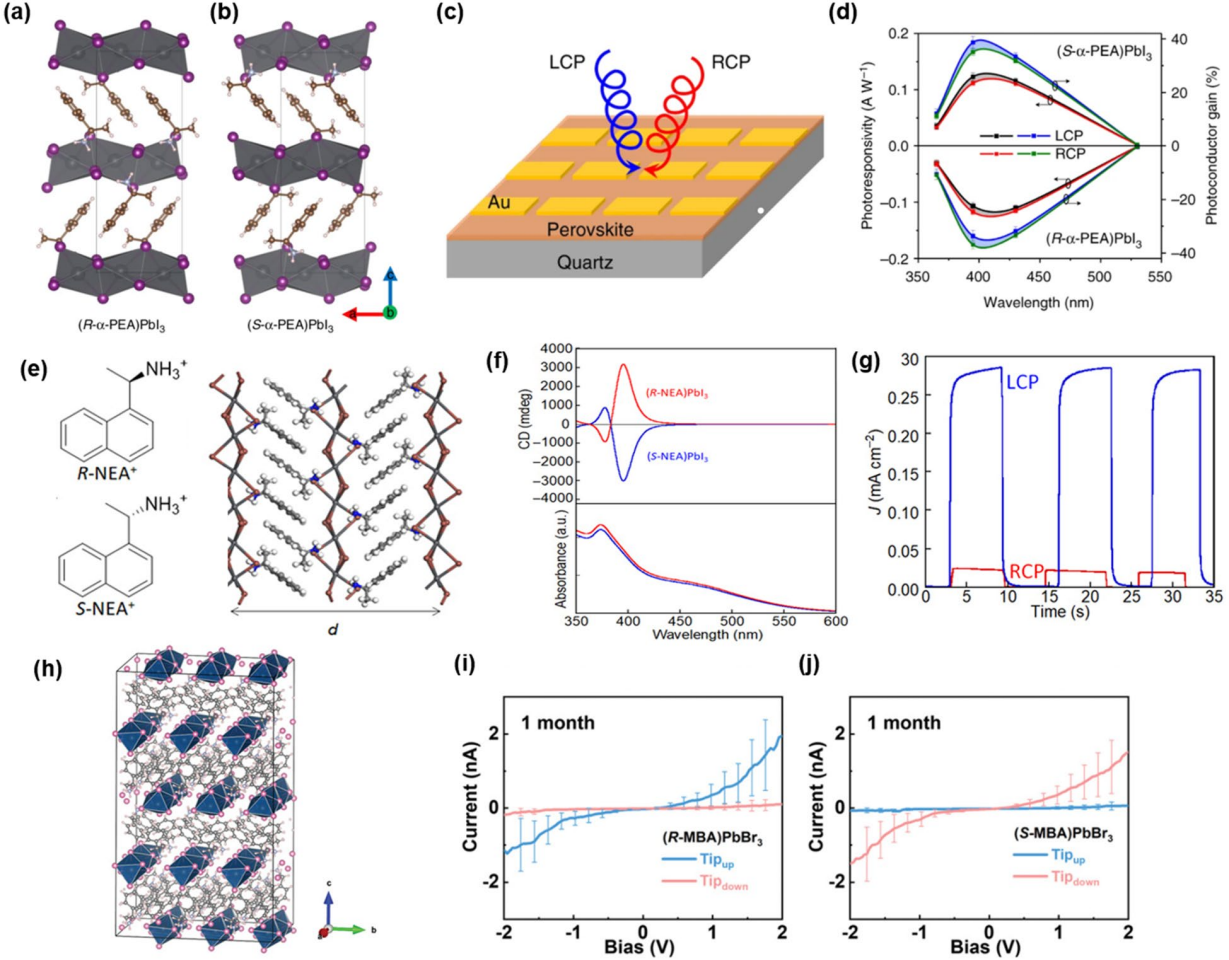
The crystal structure of **a** (*R*-MBA)PbI_3_ and **b** (*S*-MBA)PbI_3_. **c** Schematic diagram of 1D chiral (MBA)PbI_3_ based CPL detector. **d** The responsivity and photoconductor gain of 1D chiral HOIPs with opposite handedness under LCP and RCP. Reproduced with permission from [38]. Copyright 2019 Nature Publishing Group. **e** The molecular structure of *R*-NEA and *S*-NEA. **f** CD and absorption spectra of 1D chiral (NEA)PbI_3_. **g** Time course of photoresponse of (*R*-NEA)PbI_3_ under LCP and RCP. Reproduced with permission from [39]. Copyright 2020 AAAS. **h** Crystal structure of (MBA)PbBr_3_. **i**, **j** Spin-dependent charge transport *I-V* curves after 1 month. Reproduced with permission from [40]. Copyright 2021 Wiley

Jiang Tang's group reported the circularly polarized light (CPL) detection device with 1D chiral HOIPs films for the first time [38]. The researchers synthesized (MBA)PbI_3_ 1D chiral HOIPs by cooling crystallization method and HOIPs films exhibited higher CD value and *g*-factor than 2D chiral HOIPs. XRD data showed that 1D chiral HOIP has a crystal structure with the strongest diffraction peak near 8° where (PbI_6_)^4−^ octahedral chains stack in parallel (Fig. 5a, b). The 1D (MBA)PbI_3_ photodetecting film showed strong CD signals along with differences in photoconductor gain and responsivity depending on the chirality of 1D chiral HOIPs (Fig. 5d).

Although Jiang Tang's group successfully built a CPL photodetector based on 1D chiral HOIPs, the polarization discrimination ratio was too low to achieve accurate CPL detection. Ishii et al*.* used *R*-(+) and *S*-(−)-1-(1-naphthyl)ethylamine (*R*- and *S*-NEA), a new type of chiral ligand with naphthalene skeleton, to synthesize 1D chiral HOIPs which show high CD and *g*-factor [39]. Due to large π–π interaction between naphthalene, (PbI_6_)^4−^ octahedral chains strongly induce large helicity (Fig. 5e). The researchers also controlled the dimensions of chiral perovskite films with the various molar ratios of NEA to Pb^2+^ in the precursor solution. 1D and 2D structures exhibit different XRD diffraction peaks and 2D grazing-incidence wide-angle X-ray scattering measurements demonstrate that 1D chain lies against the substrate differently from 2D films which are highly aligned in out-of-plane direction to the substrate. NEA-based 1D chiral HOIPs showed CD signals over 3000 mdeg with a high ***g ***factor of 0.04, which are the highest results ever reported in chiral perovskites (Fig. 5f). Finally, by using NEA-based 1D chiral HOIPs, the CPL detectors performed an extremely high polarization discrimination ratio of 25.4, which is the highest value compared to chiral plasmonic metamaterials and organic materials-based CPL detectors (Fig. 5g).

The stability of the device is an important factor for practical applications. Lu et al. reported (MBA)PbBr_3_ 1D chiral HOIPs with high stability [40]. Unlike iodide ions, bromide has a lower Fermi level which helps (MBA)PbBr_3_ to maintain high crystallinity even in the ambient environment and high temperature. The researchers synthesized Br-based 1D chiral HOIPs with a cooling process and then confirmed the high crystallinity of the film with XRD diffraction measurement. The CISS of 1D chiral (MBA)PbBr_3_ is examined by mc-AFM and the opposite feature of *I-V* curves confirms the spin polarization of the films. The air-stability and thermal stability were analyzed with the one-month-old sample and the results showed that spin polarization efficiency and crystallinity were maintained. In summary, these results suggest that (MBA)PbBr_3_ show similar spin-polarized charge tunneling behavior and higher stability which can solve the current limitations of chiral perovskites (Fig. 5i, j).

## Magnetic properties of chiral NMs

Unlike chiral inorganic NMs with unique electronic or optical properties, the family of chiromagnetic NMs remained largely uncharted [41]. One of the reasons would be the impact of size on chiral properties and magnetic properties that these NMs possess. Like other NMs, chiral NMs exhibit their unique properties due to their high surface-to-volume ratio maximizing chiral interactions with the surrounding [42]. The magnetic performance of NMs does not increase as NMs size decreases. Still, NMs magnetic properties reach their maxima when the size falls within a specific range which differs according to the composition and morphology [43, 44]. These complex relationships in NM size might have veiled the field of chiromagnetic NMs. Considering that magnetic properties are governed by the crystal symmetry whilst introduction of chirality into NMs may break their pristine symmetrical elements, it is convincing that the area of chiromagnetic NMs still remain challenging yet intriguing [9, 45, 46].

**Fig. 6:**
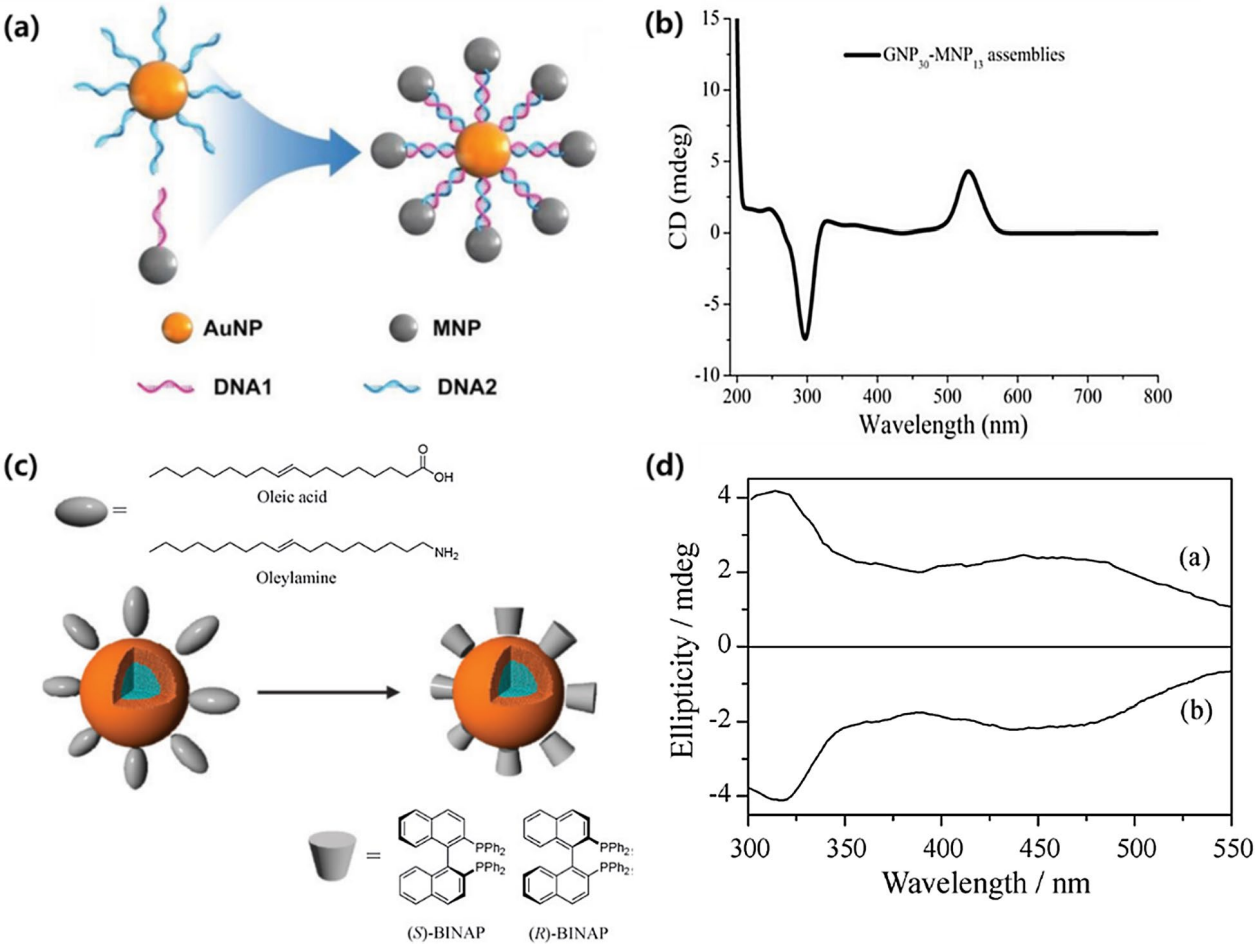
**a** Schematic representation of gold-magnetite core-satellite chiromagnetic nanostructures and **b** its CD spectra. Reproduced with permission from [47]. Copyright 2021 Wiley. **c** Schematic representation of core (iron oxide, blue)-shell (palladium, orange) chiroptical NPs conjugated with (*S*)-/(*R*)-BINAP and **d** their CD spectra. Reproduced with permission from [48]. Copyright 2009 Royal Society of Chemistry

One of the proposed methods to overcome the limitation of current examples of chiromagnetic NMs is utilizing a metal–metal oxide hybrid structure. Wu et al. introduced gold-magnetite core-satellite chiromagnetic nanostructures bridged by double-stranded DNA molecules [47]. In the study, plasmonic gold NP assembled by chiral DNA linkers (Fig. 6a) showed distinctive CD spectra (Fig. 6b), while simple mixtures of each building block were chiroptically silent. The authors attributed the result to a combination of charge transfer through chiral ligands and the typical chiral plasmonic signal of gold nanoparticles (NPs). Mori et al. reported core–shell chiromagnetic NPs with iron oxide core and palladium shell conjugated with chiral 2,2′-bis(diphenylphosphino)-1,1′-binaphthene (BINAP) molecules (Fig. 6c) [48]. The authors successfully synthesized the chiromagnetic oxide core-metallic shell NPs with mirrored CD spectra (Fig. 6d) through simple ligand exchange procedures. It was proven that the chiromagnetic core–shell NPs could be applied for chiral catalysts during the synthesis of binaphthalene with enantioselectivity of around 47%.

Unlike noble metal-based chiral NMs that can be synthesized with various methodologies [2], magnetic metal oxide-based chiral NMs mainly formed by chiral ligand-assisted growth of inorganic cores with distinctively twisted atomic arrangements [49, 50]. Interestingly, the chirality of atomic arrangements and structures strongly determines the chiroptical activity and magnetic properties of the chiral metal oxide magnetic NMs [46].

**Fig. 7:**
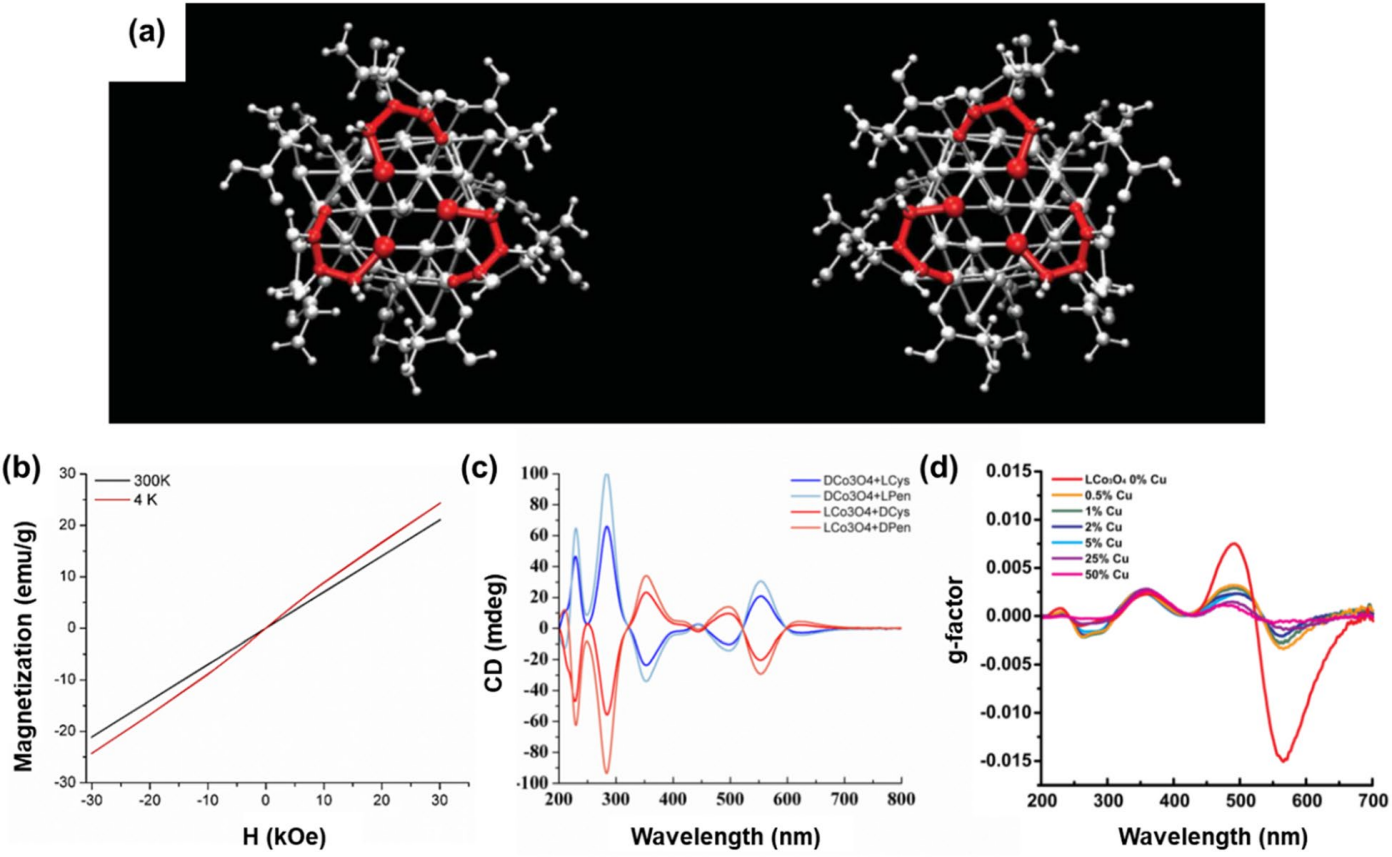
**a** d-Cys and l-Cys Co_3_O_4_ NPs. **b** Magnetic hysteresis loop at 300 K and 4 K. **c** CD spectra of d- and l-Cys Co_3_O_4_ NPs with their ligand-exchanged counterparts using l- and d-penicillamine. **d** *g-*factor spectra of l-Cys Co_3_O_4_ NPs with varying their Cu atomic concentration. Reproduced with permission from [9]. Copyright 2018 AAAS

In pristine achiral cobalt oxide (Co_3_O_4_) NPs, an ensemble of normal spinel structures and electronic configurations of cobalt cations in octahedral and tetrahedral sites contributes to the antiferromagnetic behavior of the NPs according to the crystal field theory [46, 51]. However, when l- and d-enantiomers of amino acids (cysteine, Cys) were used during the synthesis as chiral ligands (Fig. 7a), the chiral cobalt oxide NPs exhibit paramagnetic behavior instead of typical antiferromagnetic behavior above and below the Néel temperature (Fig. 7b) [9]. Because the magnetic properties of cobalt oxide are governed by its crystal lattice and atomic ordering [51], the magnetic property modulation of the chiral cobalt oxide NPs is attributed to crystal lattice distortion and symmetry breaking, both of which are induced by chirality infusion. The ab initio molecular dynamics (AIMD) calculation confirmed that the structural symmetry breaking was triggered by chiral ligands on the surface. This study also demonstrated that the control over magnetic properties using chirality of the structure is applicable to other transition metal oxides, such as nickel(II) oxide (NiO) NPs, whose pristine achiral NPs also exhibit antiferromagnetic behavior owing to their cubic rock-salt structure [9, 52].

In addition to the different magnetic behaviors exhibited by chirality, it is also worthwhile to demonstrate how the magnetic moment of the chiral NPs affects its opto-magnetic interactions with a radiated circularly polarized electromagnetic field. Several studies demonstrated that optical activity of chiral metal nanostructures is predominantly governed by localized surface plasmon resonance [53–55]. In contrast, for chiral nanostructures composed of transition metal oxides, the electronic configurations and magnetic moments derived from the configuration play a pivotal role in their chiro-optical properties [9, 46, 56]. In the case of the above-mentioned chiral Co_3_O_4_ NPs, CD spectra obtained from l-Cys-coated NPs and d-Cys-coated counterparts exhibited highly mirrored features containing eight distinctive peaks from the ultraviolet (UV) region (200 nm) to the visible range region (700 nm) (Fig. 7c) [9]. The authors of this study attempted to unravel and clarify the causality between chiral structural factors of NPs and their distinctive CD spectra, including ligand and composition exchange. For ligand exchange using l- and d-penicillamine (Pen), instead of the original chiral ligands (l-/d-Cys) after the formation of the NPs, the CD spectra in the UV range changed. In contrast, the spectra for the visible range were left undisturbed (Fig. 7c). The fact that the structural chirality of chiral cobalt oxide NPs was preserved after their surface chiral ligands were removed infers that the chiral ligand contributed to the UV range of the CD spectra while the visible range of the spectra was governed by the structural chirality of the inorganic Co_3_O_4_ cores [9, 57]. When it comes to the partial composition replacement of the inorganic cores using copper cation (Cu^2+^) instead of cobalt cation (Co^2+^), a decrease in optical activity across the visible range was detected (Fig. 7d) [9]. Considering the electronic configuration of copper cations located at tetrahedral sites where only a single unpaired electron per ion exists, magnetic moments that contribute to the chiro-optical properties of inorganic cores would decrease compared to the unchanged chiral Co_3_O_4_ NPs, which leads to a decrease in the optical activity of the NPs [58]. This explanation is supported by the fact that the reduced optical activity was restricted across the visible range which originated from the inorganic core.

**Fig. 8:**
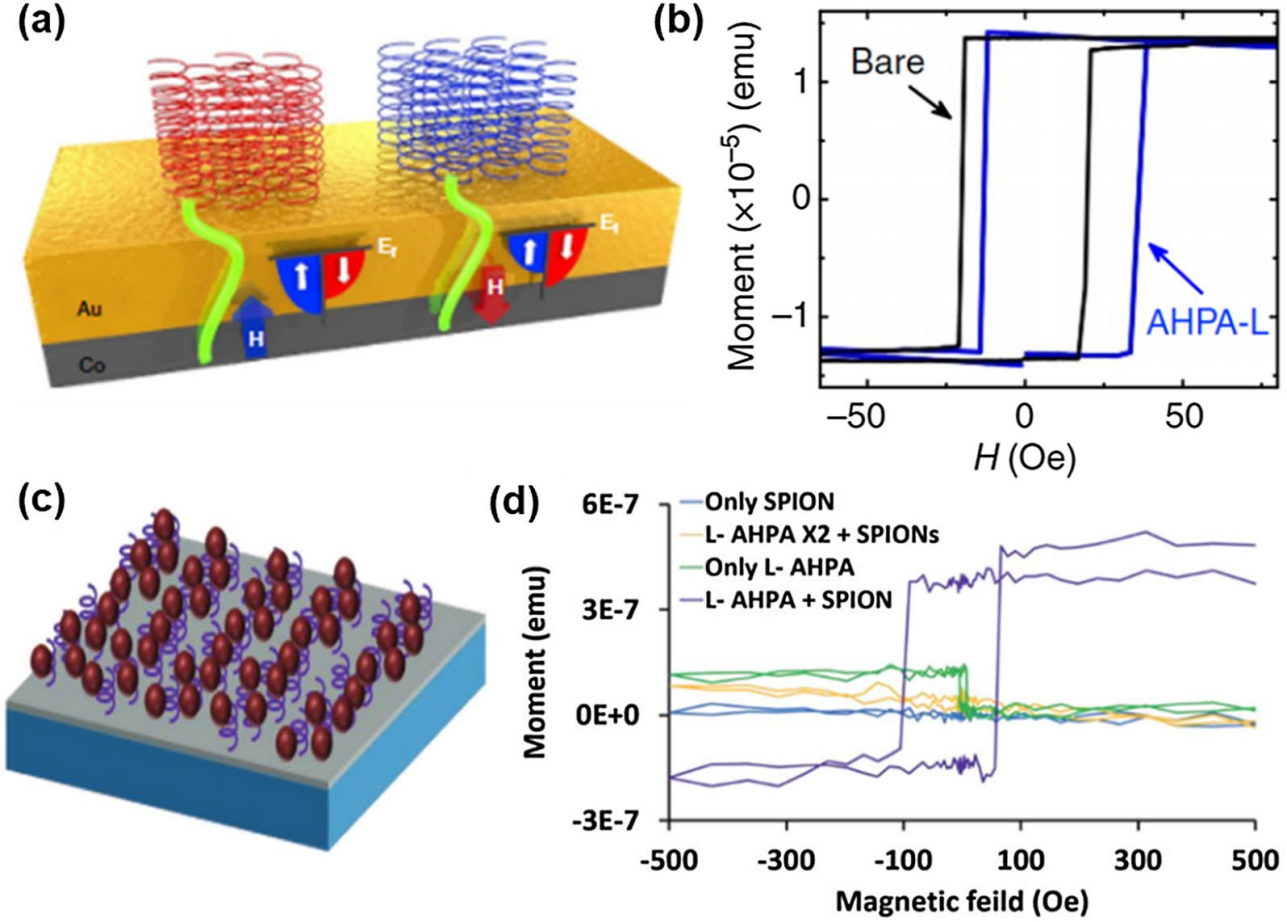
**a** Schematic representation of samples with shifted hysteresis loop using metallic cobalt-gold bilayer with chiral polypeptide deposition. **b** Shifted magnetization hysteresis loop of l-polypeptide (l-AHPA) deposited bilayer sample compared with bare bilayer sample. Reproduced with permission from [59]. Copyright 2017 Nature Publishing Group. **c** Schematic representation of single domain Fe_3_O_4_ NPs (SPIONs) with ferromagnetism deposited on a chiral polypeptide layer. **d** SQUID measurements of magnetizations hysteresis loop. SPIONs with an l-AHPA layer exhibit hysteresis loop comparable to hysteresis loop of typical ferromagnetic NPs. Reproduced with permission from [60]. Copyright 2019 Wiley

In addition to using inorganic chiral structures to tailor the magnetic properties of inorganic NPs, the magnetic properties and their behaviors can also be manipulated by the attachment of chiral molecules onto the surface of achiral inorganic nanostructures including metal and metal oxide NPs [59, 60]. When an external magnetic field is introduced, electrons with a particular spin state escape easily, while electrons with other spin states remain in the inorganic nanostructure. The surplus of unpaired electrons can contribute to the ferromagnetic behavior of the nanostructure [60]. Using this concept, a metallic cobalt-gold bilayer with a polypeptide layer deposition exhibits ferromagnetic hysteresis loops shifted toward a specific magnetic field direction (Fig. 8a, b) [59].

Koplovitz et al. reported magnetite (Fe_3_O_4_) NPs with a single domain that exhibits ferromagnetic behavior by placing superparamagnetic magnetite NPs on a chiral polypeptide layer deposited on a silicon wafer chip (Fig. 8c, d). In contrast, pristine magnetite NPs with a single domain generally showed superparamagnetic behavior [60]. This report suggests a new hysteresis loop engineering pathway for inorganic NMs using chiromagnetic effects such as the CISS.

**Fig. 9:**
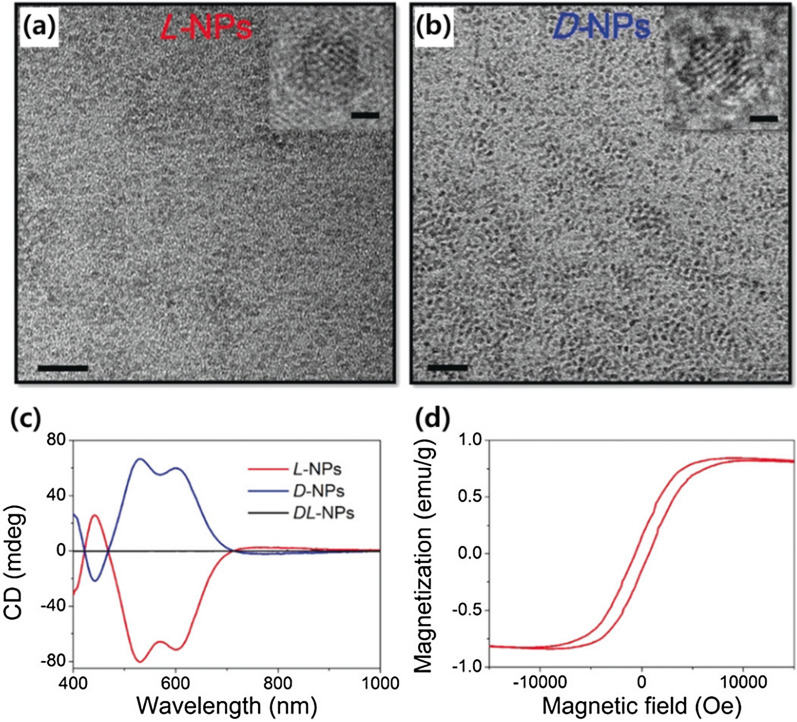
Transmission electron microscope (TEM) images of **a** l- and **b** d-penicillamine stabilized Cu_x_Co_y_S NPs, scale bar = 20 nm. The upper-right insets are TEM images of single NPs, scale bar = 1 nm. **c** CD spectra of l-/d-/dl-Cu_x_Co_y_S NPs. **d** A magnetization hysteresis loop of l-Cu_x_Co_y_S NPs. Reproduced with permission from [2]. Copyright 2020 Wiley

Li et al. adapted chiral metal sulfide NPs to study magnetic effects in nanostructures [2]. Instead of using traditional iron oxide NMs, exploiting the copper-cobalt sulfide (Cu_x_Co_y_S) NPs enabled infusion of chirality utilizing thiol-containing chiral molecules such as penicillamine whilst maintaining moderate ferromagnetism (Fig. 9). Owing to the chiral surface of the NPs, d-Cu_x_Co_y_S NPs exhibited improved cellular uptake efficiency compared with their l-type counterparts. Based on these results, the chiromagnetic copper-cobalt sulfide NPs applied to eliminate senescent cells under an alternating magnetic field with near-infrared photon illumination.

Grafting the concept of chirality onto magnetic NMs would be worthwhile for further studies due to its versatile approaches across various fields of nanotechnology. In the field of nanomedicine, theragnostic techniques rely on biocompatible magnetic NMs for magnetic particle imaging (MPI) and hyperthermia treatment [61–63]. Here, magnetic hysteresis modulation using tunable chirality is applicable for maximizing the theragnostic efficiency. Chiromagnetic modulation may also be useful for spintronics. As Naaman et al*.* addressed [5], current spintronic devices require separate layers of magnetic materials and inorganic spin filter materials. However, by integrating these layers onto a single magnetic layer, spintronic devices with both lower energy consumption and high spin selectivity can be constructed.

## Chiroptical properties of inorganic nanostructures

Chiral NMs exhibit various optical responses depending on their handedness, compositions, and scale under electromagnetic wave radiations. The unique optical properties of the chiral NMs led researchers to perform massive studies on the synthesis and fabrication methods of chiral NMs and their applications in various fields [64–68]. This chapter introduces the recent researches about chiral plasmonic NMs systems, chiral fluorescent NMs, and the interactions between circularly polarized light and chiroptically silent achiral nanostructures.

### Chiroplasmonic NMs

Chiroptical effects become particularly strong in mirror-asymmetric geometrical structures made from plasmonic NPs [1] due to strong interactions of the helical electromangnetic field of photons with the NMs with chirality in 20–200 nm scale. For this reason the chiral NMs from plasmonic materials are often referred to as chiroplasmonic. The representative optical properties of the chiral plasmonic systems are circular dichroism (CD) and optical rotatory dispersion (ORD). With the simplified model, assembly of the chiral molecule and spherical plasmonic NP, the contributions to the CD can be divided into two terms, CD = CD_molecule_ + CD_plasmon_ [69]. The plasmonic resonance enhances the CD signal of the chiral molecule, which is located in the close vicinity of the plasmonic NP surface. Along with the CD signal from the chiral molecules, new CD peaks appear in the wavelength region of localized surface plasmon resonance (LSPR) via Coulomb dipole–dipole interaction between the chiral molecule and plasmonic NP. Especially in intrinsically chiral morphologies or assemblies on chiral templates, plasmonic NMs show strong chiroptical responses by the plasmonic coupling. Thus, various fabrication methods of chiral plasmonic NMs and their applications have been extensively studied to date.

Nam's group successively synthesized 432 helicoids [70–72] showing intrinsic chiral morphologies which were induced by amino acids or peptides during seeded growth. Starting with small seed NPs with low-Miller index facets surfaces, high-Miller index facets with chiral directions are exposed on the surface as seeds grow in the growth solution. Due to the preferential binding of chiral molecules on the chiral surfaces of NPs, symmetry breaking occurs by the growth of the unblocked site toward the site blocked by chiral molecules. The helicoids have different chiral morphologies depending on the types of the seeds (cube or octahedron) and chiral molecules used (cysteine or cysteine-containing peptides).

**Fig. 10:**
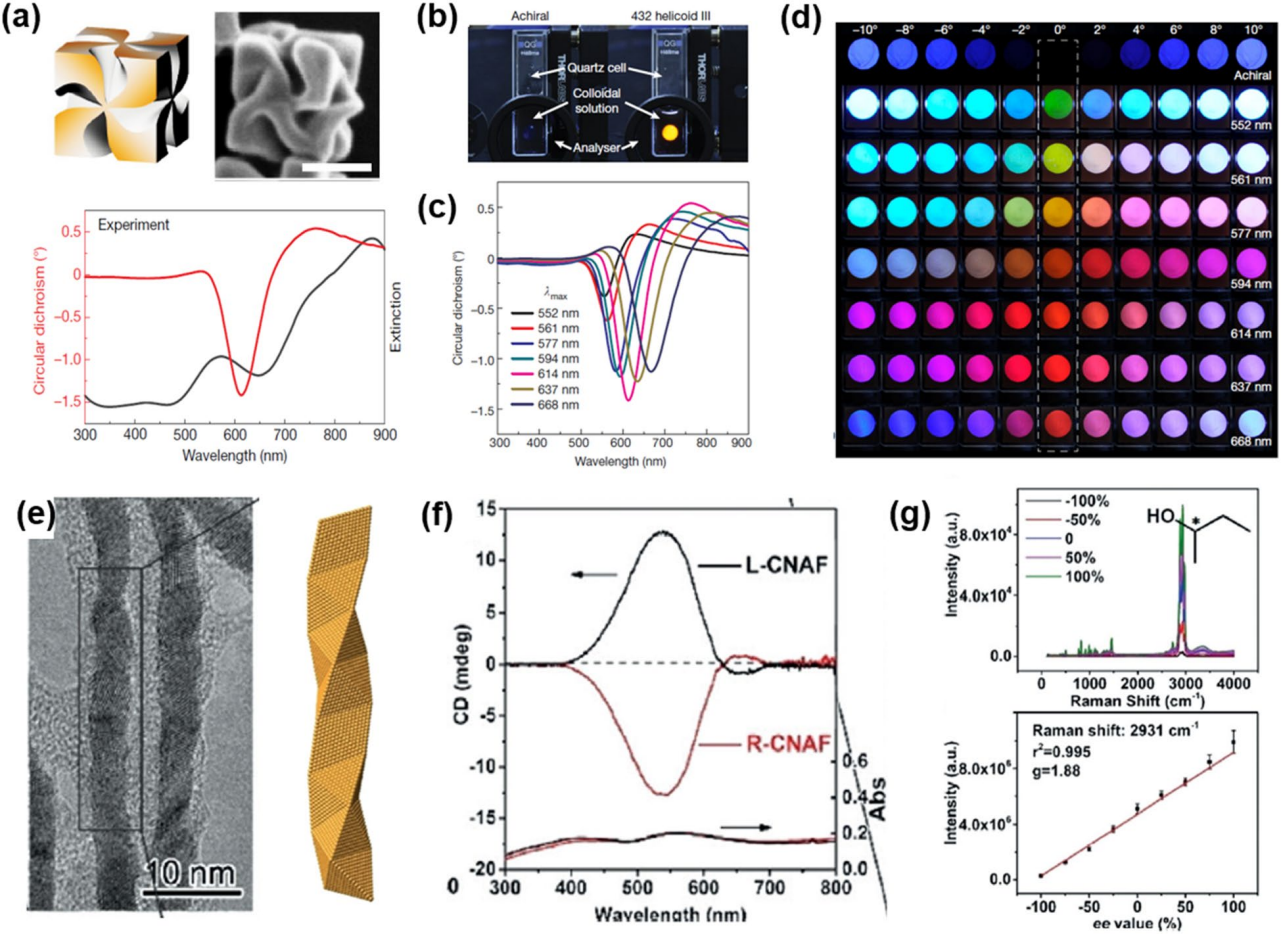
**a** 3D-model (left), corresponding scanning electron microscope (SEM) image (right), and circular dichroism and extinction spectra of 432 helicoid III. Scale bar, 100 nm. **b** Photographs of cross-polarized transmission of achiral (left) and 432 helicoid III (right) solutions. **c** CD spectra of seven 432 helicoid III dispersions having different λ_max_ values corresponding to the wavelength of maximum g-factor. **d** Polarization-resolved transmission colors of achiral nanoparticle (first row) and seven 432 helicoid III dispersions. Reproduced with permission from [72]. Copyright 2018. Nature Publishing Group. **e** TEM image of Au nanofibers with twisted morphologies (left) and representation of a right-handed chiral Au fiber with BCB helix structure. **f** Circular dichroism and absorption spectra of l- and R-chiral nanostructures Au films. **g** Raman spectra (top) and dependencies (bottom) of the largest SERS peak areas on *ee* values from − 100% to + 100% for 2-butanol, showing SERS-ChA effect of R-CNAFs. Reproduced with permission from [73]. Copyright 2020 Wiley

In particular, one of the types of chiral NPs denoted as 432 helicoid III, has a highly twisted pinwheel-like structure, which is associated with the strongest CD among the helicoids, with a *g*-factor as high as 0.2 (Fig. 10a). The authors constructed cross-polarized transmission to demonstrate the conversion from linearly to elliptically polarized light by 432 helicoid III. The achiral NP dispersion exhibited no transmission, but bright yellow cross-polarized transmission was observed from 432 helicoid III (Fig. 10b). In addition, 432 helicoid III having maximum g-factor at different wavelengths (λ_max_) were prepared for further investigation (Fig. 10c). Achiral dispersion showed symmetric and gradual color change with increasing the rotational angle of the analyzer from -10° to 10°. However, the color transition of the seven 432 helicoid III dispersed solutions with different values of λ_max_ exhibited asymmetric patterns and various transmitted colors, reflecting the optical rotatory dispersion response of the chiral plasmonic NPs (Fig. 10d). This observation indicates that the color modulation covering a wide wavelength range can be realized by unique chiroptical properties in the visible region of chiral plasmonic NMs with high asymmetric factors.

Another chiroplasmonic NM with intrinsic chirality was recently reported by Che's group with similar synthesis method, but the seeds were anchored on the 2D surfaces [73]. Surface-enhanced Raman scattering-chiral anisotropy (SERS-ChA) effect was demonstrated with normal Raman measurements of enantiomeric molecules using chiral nanostructured Au films (CNAFs) substrates. *N*-acetyl-l/d-cysteine (S/R-NAC) were used to induce chirality on Au nanofibers. During the lateral growth of the Au nanofiber on the surface of the Si wafer in the growth solution, the S/R-NAC acts as a symmetry-breaking agent by the asymmetric arrangement of Au–S, Au-NH_2_, and Au–COO bonds around the chiral carbon center. The final structure of the CNAFs is the Boerdijk-Coxeter-Bernal (BCB) structure with chiral stacking of tetrahedral units of Au nanocrystals (Fig. 10e). l- and *R*-CNAFs exhibit CD signals with opposite signs and plasmonic absorption at about 514 nm (Fig. 10f).

The normalized difference in SERS intensities of enantiomeric molecules refers to *g*-factor, g_SERS-ChA_ = 2(I_S_-I_R_)/(I_S_ + I_R_), where I_S_ and I_R_ are the SERS intensities from *S*- and *R*- enantiomers respectively. The maximum absolute value of *g*-factor is 2, which indicates complete discrimination of enantiomers. The authors measured the largest SERS peak areas of enantiomers along with enantiomeric excess (*ee*) values from − 100% to + 100% at the same concentration of analytes and plotted the curves. The *g*-factors were also obtained from *g*_SERS-ChA_ = 2 k/b, where k and b are the slope and intercept of the curve, respectively. For example, the SERS intensities of the 2-butanol enantiomers were proportional to ee values, with a *g*-factor of 1.88 (Fig. 10g). Other enantiomers also showed a linear correlation between SERS intensities and ee values and *g*-factors ranging from 1.34 to 1.99. These results indicate that the CNAFs platform has a high discrimination capability of enantiomers. The SERS-ChA phenomenon can be explained by the local chiral electromagnetic fields on the CNAF structures. The CNAFs generate asymmetric local fields around them when illuminated by unpolarized or linearly-polarized laser with the wavelength at the plasmonic absorption and CD peak. The chiral local fields enhance Raman scattering of both enantiomers, but much stronger enhancement occurs when matching with the chirality of the analytes. By the novel chiroptical property of chiroplasmonic nanofiber, SERS-ChA effect, The CNAFs platform can efficiently discriminate the racemates of enantiomers compared to the traditional techniques such as circular dichroism or Raman optical activity.

**Fig. 11:**
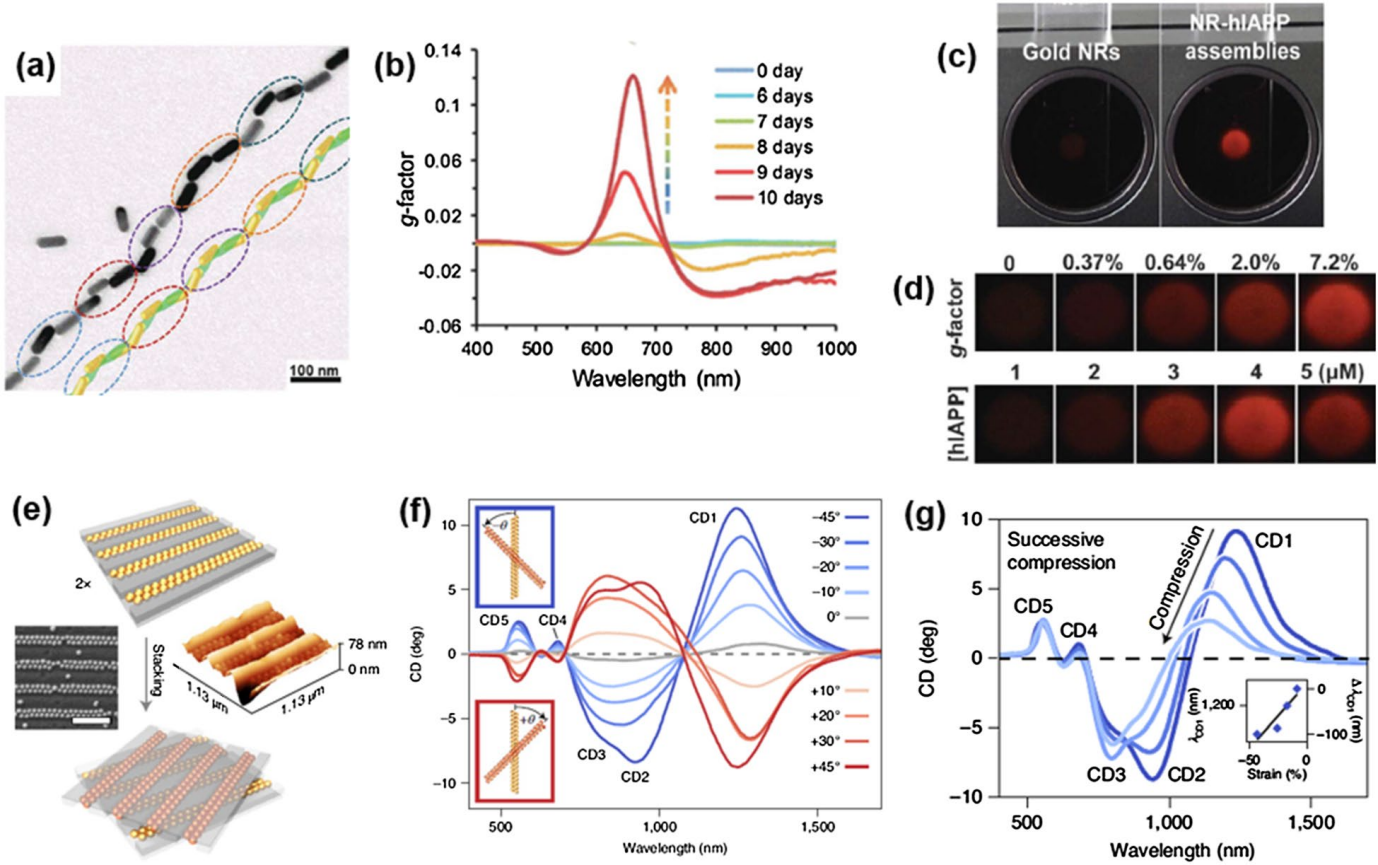
**a** TEM image of NR-hIAPP helical assembly and reconstructed model from the TEM image. Pitch of the nanohelices were represented by the circles. **b** g-factor spectra for the coassembly process of hIAPPs with AuNRs. The g-factor reached to 0.12 after the coassembly completed. **c** Photographs of the red light transmitted through pure NR (left) and NR-hIAPP assemblies (right) under cross-polarized conditions. **d** Photographs of the red light transmitted through NR-hIAPP assemblies with different g-factors (top) and coassembled from various hIAPP concentrations (bottom). Reproduced with permission from [15]. Copyright 2021 AAAS. **e** Schematic image of stacking process and SEM and AFM height images of AuNP assembled into dimer chains inside nanochannels. Scale bar, 500 nm. **f** CD spectra for the chiral metasurfaces with stacking angles from  ± 45° over ± 30°, ± 20° and ± 10° to 0°. **g** CD spectra for the chiral metasurfaces stacked at − 45° under compression normal to the bilayers. Reproduced with permission from [74]. Copyright 2021 Nature Publishing Group

Assembly of achiral plasmonic NPs into chiral arrangement can also induce strong chiroptical activities. Chiroplasmonic assemblies with increased *g*-factors were recently demonstrated by Liu's group [15]. The ensembles of the low *g*-factor constituents, AuNRs and human islet amyloid polypeptide (hIAPP), form helical conformation with LC-like long-range order and the end-to-end oriented long-chain NRs (Fig. 11a). hIAPP is bound on the AuNR via Au–S bond between cysteine residue of hIAPP and AuNR in the long-range registry of the assemblies. The cetyl-trimethylammonium bromide (CTAB) bilayer on the surface of AuNRs enhances the fibrillation of hIAPPs and strengthens supramolecular attractions between peptides. Consequently, the CTAB- capped NRs accelerate the helical self-assembly of hIAPP compared to hIAPP alone in dispersion.

In addition, the long-range organization of NRs on hIAPPs exhibited strong chiroptical properties with CD of 2000 mdeg and *g*-factor of 0.12 (Fig. 11b). The enhanced *g*-factor is much higher than that of ultraviolet region of the spectrum for biomolecules and the accelerated assembly provide precise and rapid sensing ability under low peptide concentration. Under the cross-polarization condition, a dispersion of assembled NR-hIAPP nanohelices made the red light at the maximum *g*-factor transmitted to be vividly observed (Fig. 11c). This is due to the optical rotatory dispersion response of the chiral suprastructure. The intensities of transmitted light increase with a higher *g*-factor of the dispersion. At the different hIAPP concentrations, the intensities of transmitted light are changed, providing the method to quantify the formation of amyloid fibers from peptides (Fig. 11d). On the contrary, the amyloid fiber inhibitors were tested to exploit the drug discovery protocols. With the increased ratio of the drugs to hIAPP, the bright red transmitted lights are darkened due to the dissociation process of the helical assemblies. The high *g*-factor- enabled imaging platform can be utilized for peptide sensors or drug discovery in biological environments by visualization of the formation or dissociation of the amyloid fibers.

Apart from the chiral assembly of plasmonic NPs with a biological templates [1, 15], chiral metasurfaces via assembly of colloidal AuNPs having high mechanical tunability were introduced by Fery's group [74]. Spherical AuNPs are assembled in the nano-channels of the elastomeric template with close-packing. Two layers of the parallel arrangement of AuNP dimer chains are cross-stacked at an angle *θ*, to induce chiroptical properties (Fig. 11e). The strong chiroptical response of the system stems from the plasmonic coupling by small inter-particle and inter-layer distances.

With the convenient reconfiguration by changing the stacking angle and inverting the handedness, the CD signals can be easily modulated in the amplitude and sign from positive to negative (Fig. 11f). The CD also can be modulated by the mechanical compression applied to the elastic template. Successive compression induces a large gradual blue shift of the CD1 and CD2 modes (Fig. 11g). The shifting of CD signals occurs because the strain-induced bending makes the AuNPs deviated from the initial position and, therefore, reduces the inter-particle plasmonic coupling. The reversible CD tunability by restacking or mechanical strain promotes the exploitation of spectroscopic devices or light modulators. For the other practical application of the system, the well-known protein, bovine serum albumin (BSA) was deposited between the substrates stacked at the angle of ± 45°. The BSA shifted the CD1 modes ΔΔ*λ* = Δ*λ* − 45° − Δ*λ* + 45° = 40 nm in NIR region, which is a higher amplitude than previous research [75]. The strong superchiral fields in the inter-layer gap enhance the detection sensitivity so that the chiroplasmonic system can potentially be used as a chiral sensor.

### Chirofluorescent NMs

Circularly polarized photoluminescence (CPP) is another unique phenomenon that characterizes chirality. For chiral molecules, it is based on the differential spontaneous emission of left and right circularly polarized radiation by luminescent systems. For NMs, circular polarization of emitted light also includes differential light scattering by the chiral nanostructures [76]. The cumulative emission dissymmetry factor can be calculated as

$${g}_{lum}=\frac{2({I}_{L}-{I_{R}})}{{(I}_{L}+{I_{R}})}$$

where *I*_*L*_ and *I*_*R*_ represent the photolumniescent intensity of LCP and RCP, respectively [77]. Differential emission can be observed at both atomic and molecular level under various conditions and can be originated from various radiative relaxation processes. For instance, CPP can be induced by the incorporation of chiral molecules in the framework [38, 78], attachment of chiral ligands on the surface of NPs [79–82], or growth of chiral superstructures [83, 84]. This section, however, will particularly focus on chirality transfer from chiral ligands to the photoluminescent core. Chiral ligands can either be directly introduced during the synthesis or be post-synthetically exchanged [82]. Polarized light is emitted from the resulting optically active chiral nanostructures.

**Fig. 12:**
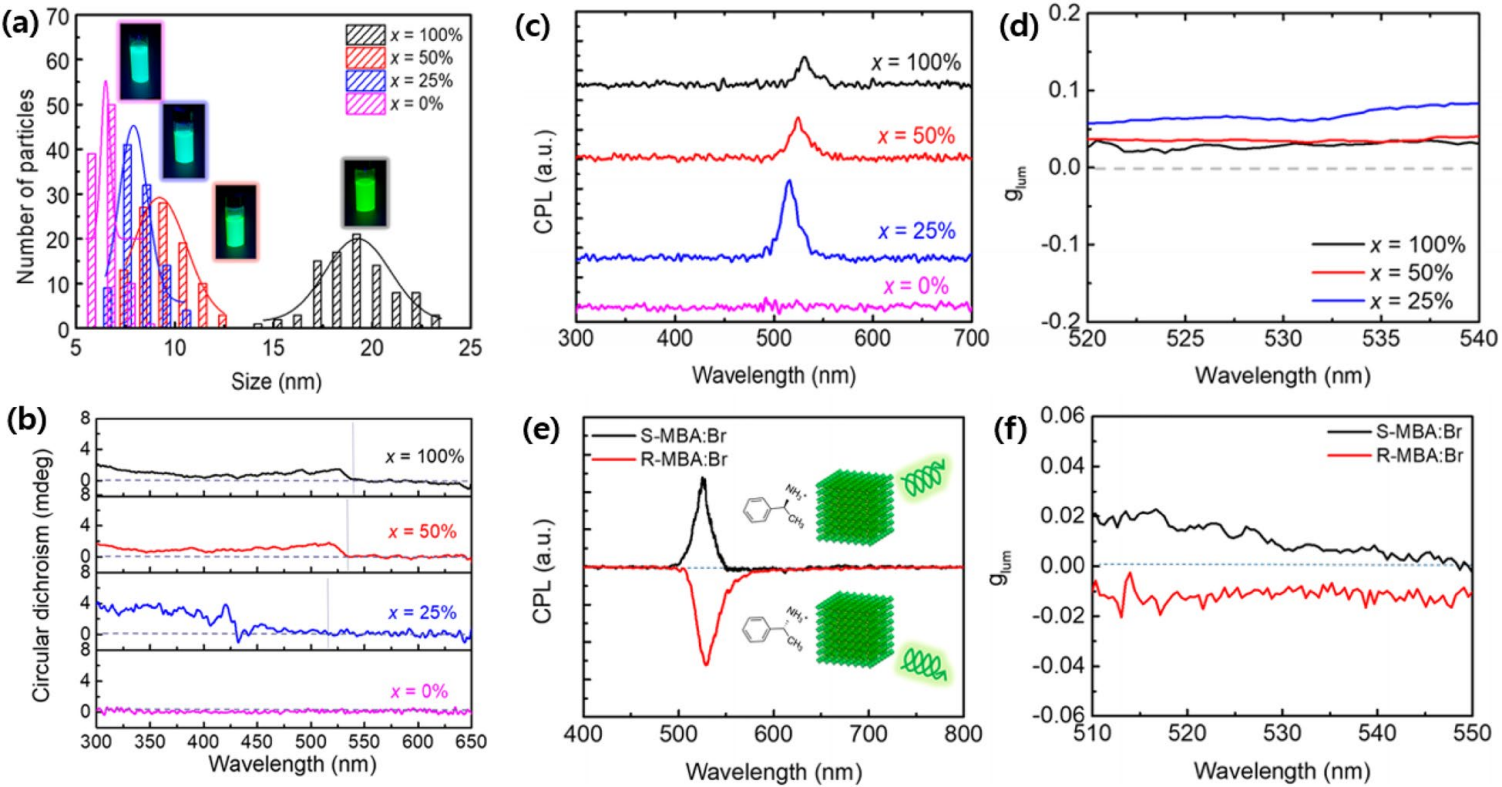
**a** Size distribution histograms and photographs under a λ = 350 Xe lamp (inset), **b** circular dichroism (CD) spectrum, **c** CPP, and **d** average glum values measured at 520 ≤ λ ≤ 540 nm of FAPbBr_3_ NP dispersion with different (*R*)-2-octylamine concentration *x*, **e** CPP and **f** average glum of ligand-treated FAPbBr_3_ NPs with *R*-,*S*-, MBA:Br. Reproduced with permission from [82]. Copyright American Chemical Society, 2020

CPP spectrometer is often utilized to investigate the geometric effects on ligand-induced chirality because such chirality is highly dependent on the size, morphology, and surface chemistry of the NPs. As chirality is induced from the synthesis stage, CPP can also provide critical information about the shape evolution and ligand-particle interactions during NP formation [81]. Young-Hoon et al. demonstrated a very high CPP by directly introducing chiral ligand during synthesis. High CPP response was achieved from colloidal formamidinium lead bromide (FAPbBr_3_) NPs at room temperature using chiral surface ligands (*R*)-2-octyalmine. The *g*_lum_ of 6.8 × 10^–2^ was achieved, which is the highest among reported perovskite materials at room temperature. FAPbBr_3_ NPs were synthesized using the hot injection method and the ratio of (*R*)-2-octylamine to oleylamine (OAm) was varied. As (*R*)-2-octylamine concentration decreased from 100 to 0%, the size of NPs decreased [82]. Figure 12a shows the size distribution of NPs depending on the concentration of (*R*)-2-octylamine. The size of NPs is determined by the steric hindrance of surface ligands; long-chain ligands (OAm) have higher steric hindrances and thus prevent diffusion of precursor species to the surface of NPs to impede NP growth [85]. Photoluminescence quantum yield (PLQY) is higher for larger NPs because an increase in surface-to-volume ratio results in more nonradioactive recombination at surface defects [82].

On the other hand, CPP intensity is strongest at a relatively low concentration (*x* = 25%). Figure 12b–d shows CD, CPP and *g*_lum_ spectra of NPs with different (*R*)-2-octylamine concentration, respectively. The results demonstrate that the absolute amount of injected chiral ligands is not the only factor in determining chiroptical properties in NPs. As smaller NPs are formed with lower concentration of (*R*)-2-octylamine, the surface-to-volume ratio increase and hence more chiral ligands can attach to the surface. Also, smaller NPs have a smaller distance between surface chiral ligands and electronic states in the core NPs, thus inducing larger effects of chiral ligands. Such incorporation of chiral organic molecules into organic–inorganic perovskite NPs provided a direct interaction between the ligand and the photoluminescent core, which is impossible in core/shell structure [82].

However, CPP diminished after NPs were purified as a significant amount of chiral ligands were removed. Loss of CPP after purification limits the application of NPs to optoelectronic devices because further purification is necessary for the formation of the high-quality thin film [86]. To overcome such a problem, Young-Hoom Kim et al. carried out post-synthetic ligand treatment by mixing a dispersion of purified NPs with a saturated solution of the chiral ligand (*R*-,*S*-MBA:Br) in ethyl acetate (EtOAc). Figure 12e, f show the restored CPP and *g*_lum_ of synthesized NPs. *R*-,*S*-MBA:Br attached on the surface of perovskite NPs enhanced *g*_lum_ up to 1.18 $\times$ 10^–2^. PLQY is also restored from 53 to 71%. The result indicates that chiral ligands passivated the surface defects and thus reduced nonradioactive recombination [82].

**Fig. 13:**
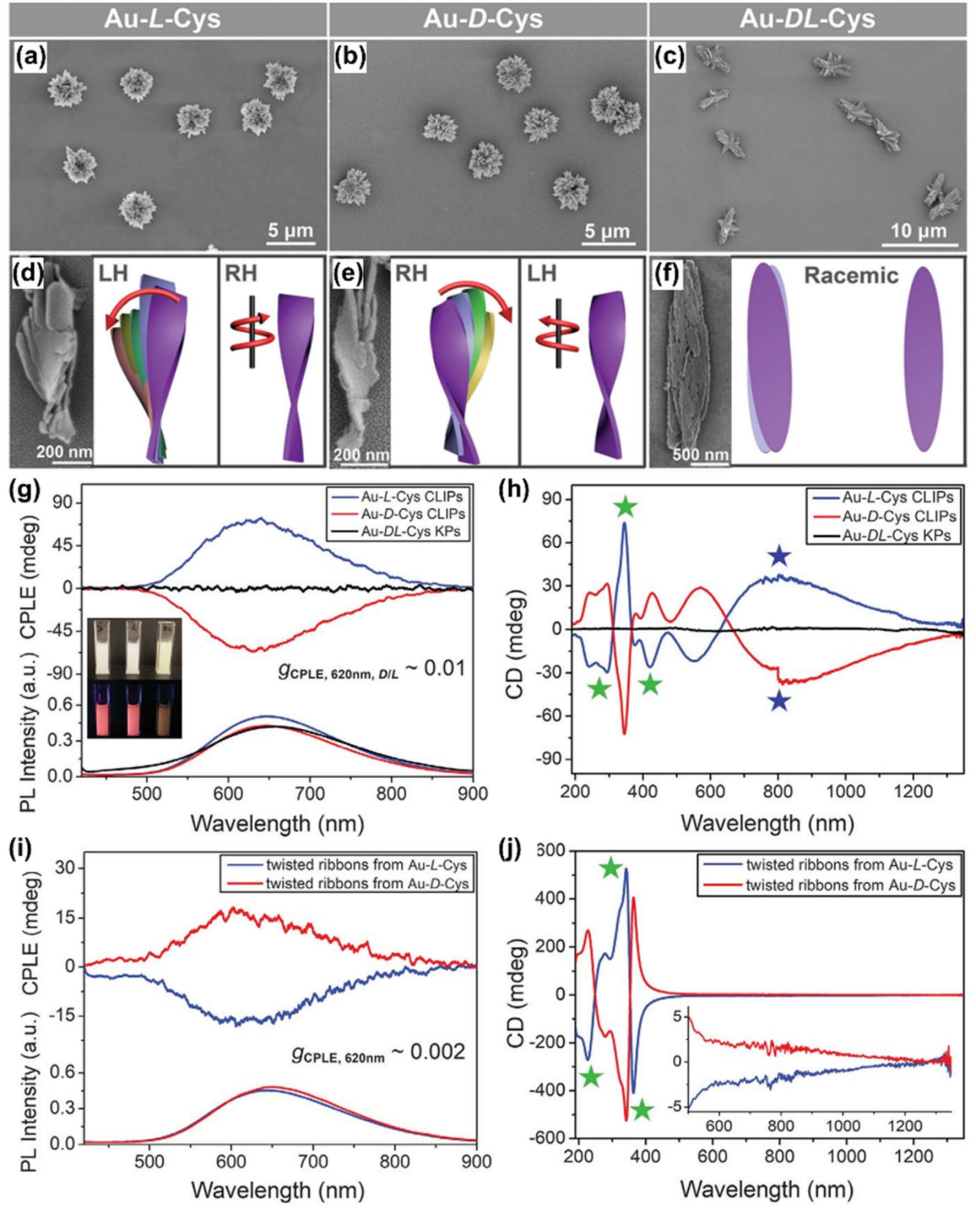
Au thiolate hierarchically organized particles (HOPs) with Cys ligands. **a**–**c** SEM images of Au–l-Cys and Au–d-Cys coccolith-like particles (CLIPs) (**a** and **b**) and Au–*DL*-Cys kayak particles (**c**). **d**–**f** SEM images and corresponding schematic illustrations of segments of Au–l-Cys (**d**), Au–d-Cys (**e**), and Au–dl-Cys (**f**). **g**, **h** CPP (**g**) and CD (**h**) spectra of Au–l-Cys CLIPs (blue), Au–d-Cys CLIPs (red), and Au–dl-Cys kayak particles (black). Inset in **g** Photos of Au–l-Cys, Au–d-Cys, and Au–dl-Cys dispersions under daylight (top) and UV light (bottom) illumination. **i**, **j** circularly polarized emission (**i**) and CD (**j**) spectra of Au–l-Cys (blue) and Au–d-Cys (red) after sonication. Inset in **j** The same spectra for the 500- to 1350-nm spectral window to confirm the absence of the CD peaks associated with differential scattering of assembled CLIPs. The helicity of the nanoribbon stacks of Au–l-Cys is left-handed, and therefore the light scattered by Au–l-Cys has left-handed polarization. After disassembly of the stacks into single right-handed nanoribbons, the light passing through these dispersions acquires right-handed circular polarization. Reproduced with permission from [12]. Copyright 2020 AAAS

Circularely polarized emission combined from CPP from chiral centers in NP and differential scattering from self-assembled mesoscale structures can be also observed in hierarchically organized microparticles made of polydisperse Au thiolate nanoplatelets. Kotov’s group synthesized hierarchically organized particles (HOPs) with the complex ordering of chiral fluorescent inorganic building blocks, in which Cys ligands were used as chiral bias during synthesis (Fig. 13) [12]. The use of pure l- and d-Cys yielded HOPs with radially organized left- and right-handed twisted spikes with a diameter of 3.5 ± 0.3 μm, which showed strong CD bands in UV–Vis and IR regions (Fig. 13a–f, h). These spiky particles were denoted as Au–l-Cys and Au–d-Cys, respectively. When a racemic mixture of Cys was used, kayak-like HOPs with layered structures, denoted as Au–dl-Cys, were observed and they were CD-silent. The HOPs had photoluminescence properties originated from atomically thin layers of Au and S connected by aurophilic bonds. Au–l-Cys and Au–d-Cys possessed red emission and with strong and variable circular polarization, whereas Au–dl-Cys showed orange emission (Fig. 13g) without helicity of emitted photons. Intriguingly, when the HOPs were disassembled into structural components (twisted ribbons) by ultrasonication, the signs of the circularly polarized emission peaks were reversed in both Au–l-Cys and Au–d-Cys (Fig. 13i, j). Comparing theoretically calculated scattering and CD spectra and experimental CD spectra, it was speculated that the circularly polarized differential scattering of HOPs is strongly involved in emission peaks because of the submicrometer-scale chirality. Thus, in HOPs the contribution of geometrical chirality (submicrometer-scale) to empirically measured CPP, which have opposite chirality in scattering photon, exceeded angstrom-scale chirality of the excited states in the Au-Cys nanosheets. However, when the HOPs were separated into the ribbons, the emission peaks mainly came from angstrom-scale chirality. These results indicated that the circularly differential polarized scattering from chiral hierarchical microstructures could influence luminescence and scattering activities, which can be altered by engineering their chiral geometry and complexity.

**Fig. 14:**
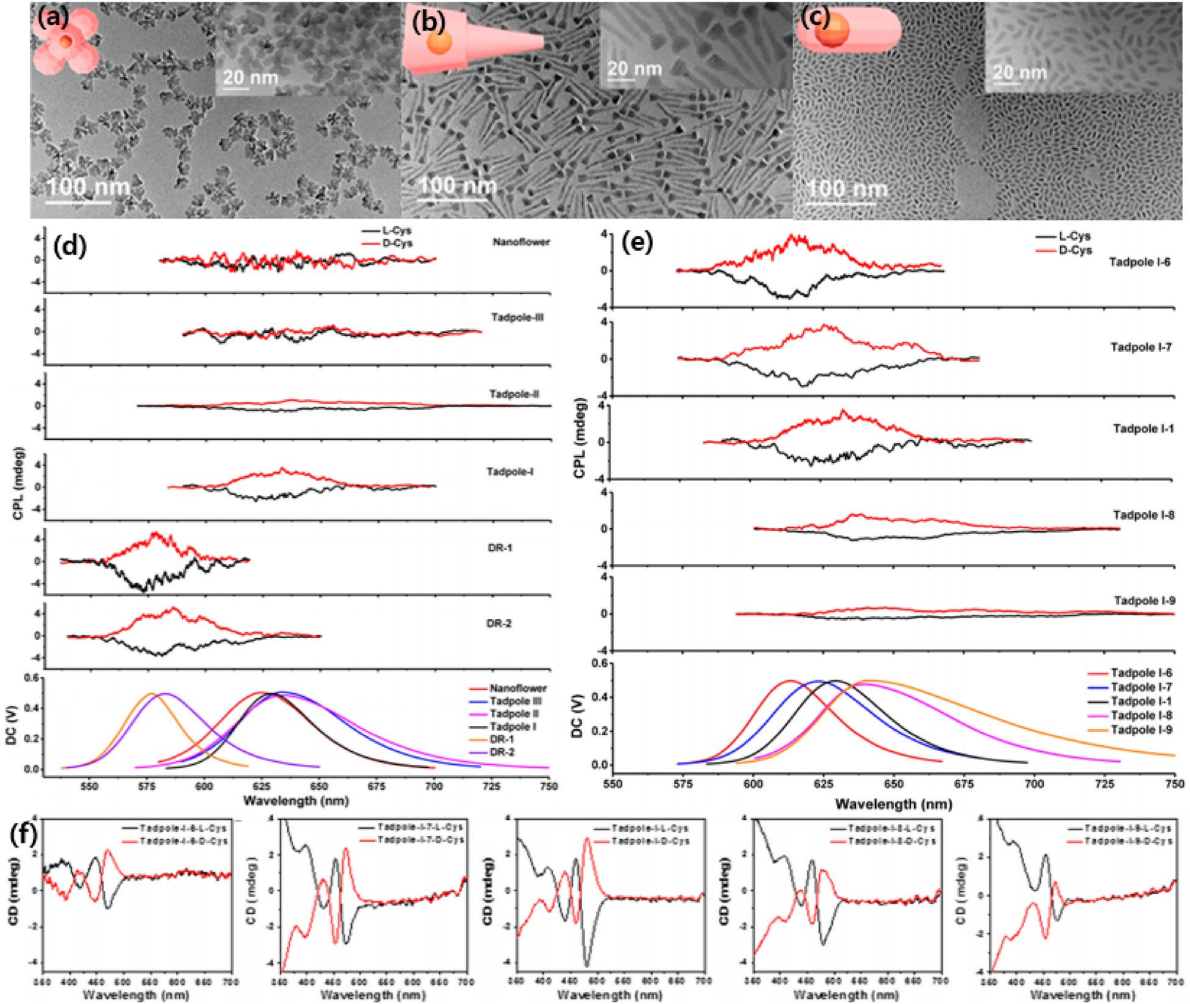
**a**–**c** TEM of CdSe/CdS NPs with different shapes: **a** nanoflowers, **b** tadpole, **c** dot/rods, **d** Empirical CPP spectra of CdSe/CdS NPs with different shapes, **e**, **f** CPP and CD spectra of CdSe/CdS tadpoles with different length. Reproduced with permission from [81]. Note that empirical CPP spectra contain increasingly high contribution from differential scattering as the NMs becomes comparable to the wavelength of light as was observed for CLIPs in Fig. 13. Copyright American Chemical Society, 2020

Another method to make NPs chiral is by post-synthetic ligand exchange. Junjie Hao et al. synthesized various types of Cys-capped CdSe/CdS nanostructures, including nanoflowers, tadpoles with one to three tails, and nanoassemblies, and demonstrated the relationship between ligand-induced chirality at the molecular level and geometrical effects at the nanoscale. Figure 14a–c shows the TEM images of CdSe/CdS NPs with different morphologies such as nanoflowers, tadpole, and nanoassemblies. Anisotropic CdSe/CdS NPs were synthesized first in the organic phase and then ligands were synthetically exchanged with l/d-cysteine. Various morphologies of NPs are controlled by adjusting the amount and chemical nature of the surface ligands [81].

CD/CPP spectrometer reveals the geometric effects, including shell thickness and length-to-diameter ratio, on the ligand-induced chirality of NP. Figure 14d shows CPP spectra of the CdSe/CdS NPs with different trends. The thickness of the shell decreases in the order of nanoflowers, tadpole, and nanoassemblies. While CPP is proportional to PLQY, PLQY of different NPs decreased as the shell thickness increased due to an increase in defects and lattice mismatch. CPP activities expressed with the *g*_lum_-factor increase in the order of nanoflowers, tadpoles, and DRs. Both CD and CPP decrease as CdS shell thickness increases. Thick CdS shell hinders interaction of CdSe core, which mainly attributes to CD signal, with chiral ligand, and hence reduces CD intensity. Likewise, a thin CdSe shell is favorable for increasing the number of chiral interactions between the CdSe cores and the chiral ligands, which determine the CPP intensity [81].

Moreover, in situ CD/CPP observation during synthesis of chiral tadpole was utilized to study growth mechanism and the tail length effect on the chiroptical responses. Figure 14e, f show the CPP and CD spectra of synthesized tadpoles with different tail lengths. While the longer tail of the tadpole induces stronger CD intensity, CPP intensity is decreased and even diminished due to the quenching effect. Although a longer tail may introduce geometrical anisotropy for CD increment, it hinders ligand chirality induction for CdSe/CdS NM and reduces emission. Therefore, the parameters to achieve the highest CD and CPP are not necessarily the same. The optimal structure to obtain multiple CPP and CD line shapes with high intensity would be chiral tadpoles with a thin shell, reasonable QY for photoluminescent core, and a moderate tail length/aspect ratio for the CdS tail [81]. Note that for some of these structures the contribution of the differential scattering in empirically measures CPP spectra can also be high.

CPP is tunable with various parameters, including size, surface chemistry, and shell thickness in core/shell structure. The optimal parameter for the strongest CPP production, however, is not necessarily the same as those for CD. With the tunable characteristics, NPs producing CPP can be utilized in various optical and biological applications including information storage and processing, quantum communication, 3D display, bio encoding, and photoelectric devices [82].

### CPL as a chiral inducing agent

Wet-chemical synthesis of chiral NMs has been achieved mainly via the surface engineering of the inorganic materials using chiral molecules such as amino acids, helical polymeric chains, and other chiral organic molecules [72, 87, 88]. However, another approach to form chiral inorganic nanostructures has been introduced to form enantioselective inorganic NMs by using CPL, inspired by the fact that photon illumination can be used for inorganic NPs self-assemblies [89, 90] due to the high optical and chemical activities of semiconducting and metallic NMs.

**Fig. 15:**
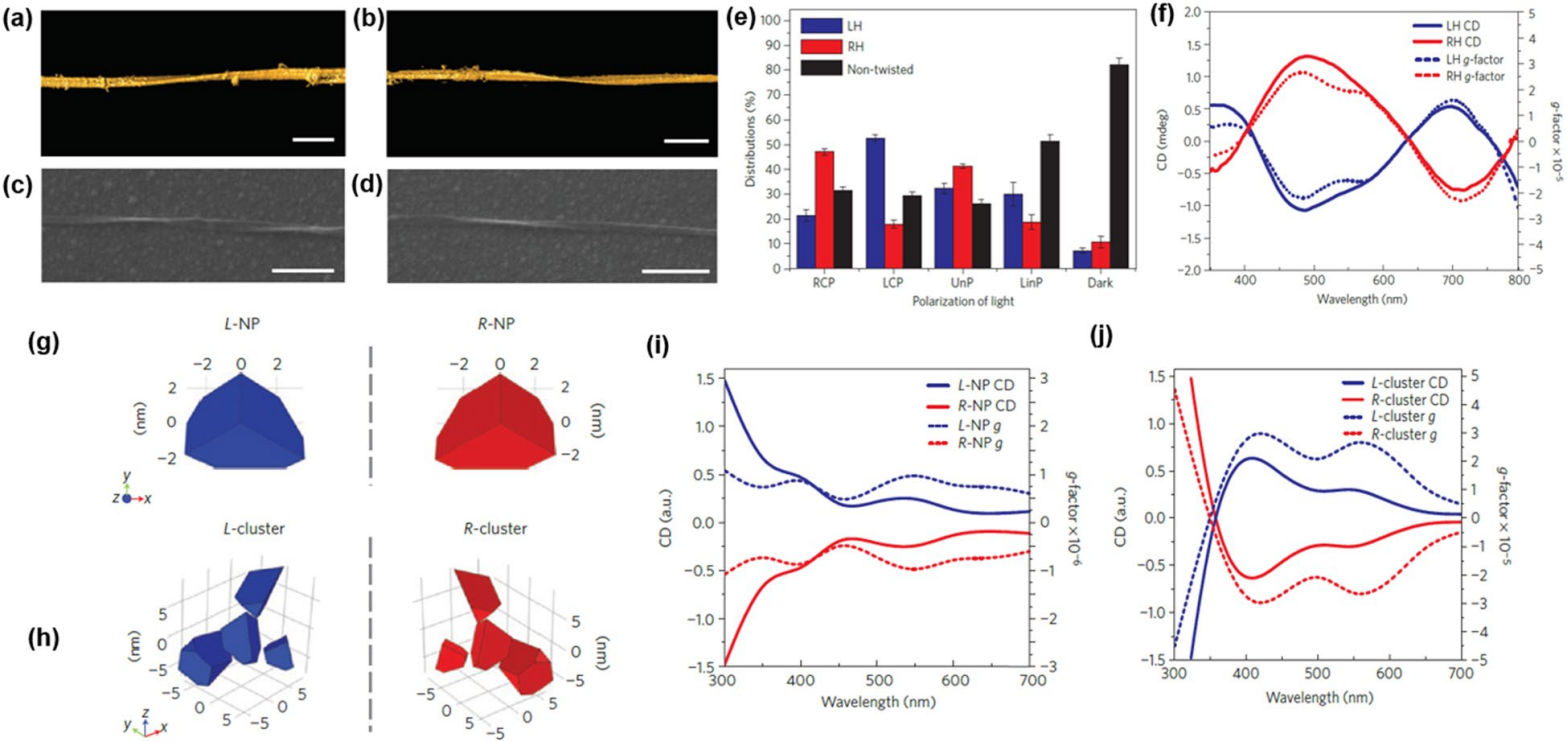
**a**, **b** 3D TEM tomography of **a** LH and **b** RH single twisted nanoribbons. Scale bars, 100 nm. **c**, **d** SEM images of **c** LH and **d** RH single twisted nanoribbons. Scale bars, 500 nm. **e** Distributions of LH, RH and non-twisted nanoribbons yielded after 50 h illumination under RCP, LCP, unpolarized light, linearly polarized light, and in the dark. **f** CD spectra (solid line) and g-factors (dotted line) of LH and RH twisted nanoribbons obtained after 50 h illumination. **g**, **h** Models of chiral NPs (**g**) and chiral NP clusters (**h**) used in calculations of the chiroptical properties. **i**, **j** Simulated spectra and g-factors for chiral NPs and chiral NP clusters. Reproduced with permission from [91]. Copyright 2015 Nature Publishing Group

Yeom et al. [91] investigated a synthesis method for chiral structured semiconductors, using CPL as a chiral templating guide. The chirality transfer of helical light to the inorganic self-assembled structure was realized by illumination of either LCP or RCP light to a thioglycolic acid (TGA)—stabilized CdTe NP dispersion, where the 543 nm wavelength of the light was chosen because of the strong absorption band of CdTe NPs in water. Twisted nanoribbon morphologies and chiroptical activity were investigated using three-dimensional (3D) TEM tomographic reconstruction, SEM images, and CD spectra (Fig. 15a–d). Left-handed (LH) twisted nanoribbons were dominant under LCP illumination, whereas right-handed (RH) twisted nanoribbons were dominant products with similar dimensions of LH twisted nanoribbons when the NP dispersion was illuminated with RCP with over 30% enantiomeric excess (Fig. 15e). In addition, the CD spectra of the two batches had mirror-imaged CD spectra, which exhibited distinct chiroptical bands at 490, 590, and 700 nm (Fig. 15f). Unlike the circularly polarized light, the illumination of unpolarized light illumination formed a racemic mixture of RH and LH twisted nanoribbons and there were no detectable CD activities.

The mechanism of the formation of the permanent shape with opposite handedness is understood as follows. First, the photochemical reaction caused the CdTe NPs to self-assemble. The illumination of light-induced photo-oxidation of TGA and replacement of Te^2−^ ions by S^2−^ ions transforms the CdTe NPs into bare CdS NPs. The ligand-free CdS NPs are self-assembled more easily than the ligand-attached and non-photo-activated CdTe NPs. Second, the light absorption efficiency difference between l-CdTe NPs and d-CdTe NPs made the predominant handedness according to the light handedness. The enantioselective optical absorption difference at 543 nm was revealed by the simulation of truncated tetrahedron NPs and their clusters (Fig. 15g, h). The l-NPs and l-clusters exhibited positive CD signals; on the other hand, the d-NPs and d-clusters showed negative CD peaks **(**Fig. 15i, j). From this point of view, when a racemic mixture of chiral CdTe NP suspension is illuminated with LCP, the LH-NPs absorb light more efficiently than RH-NPs, which increases the formation of LH twisted nanoribbons predominantly and vice versa.

Owing to its simplicity and universality, light-driven synthesis of chiral nanostructures is also applicable to metallic materials. Plasmonic NPs are promising candidates for photon-to-matter chirality transfer because highly delocalized plasmonic states in metals provide strong rotary power [11]. The handedness of helical light can influence the growth and assembly of metallic NPs. For instance, hot-electron processes are likely to be both spin- and site-selective, leading to asymmetric particle growth. Plasmonic coupling and hydrodynamic fields may influence the assembly process, thus making it light-handedness-dependent.

However, light-driven synthesis is more challenging for metals than for semiconductors because the lifetime of excited states is shorter, and thus the probability of photoinduced reactions is reduced. Moreover, it is difficult to investigate the chiral optical properties of metallic materials. While organic materials show easily distinguishable chiral shapes, such as helices or tetrahedrons, metallic NPs show non-obvious handedness. At the nanoscale level, metallic NP assembly may show geometrical complexity and a seemingly achiral structure, even when they are not. In addition, CD peaks are polysemous for inorganic nanostructures because multiple optical processes contribute to their optical activity [11, 92–94]. Therefore, careful analysis of chirality at nanoscale dimensions is crucial for interpretation.

Despite these challenges, the synthesis of NPs and their subsequent assembly into chiral nanostructures have been demonstrated by the illumination of gold salt solutions with circularly polarized light [18]. An aqueous solution of Au(III) chloride hydrate and citrate was irradiated under LCP or RCP light at a wavelength of 543 nm. Red dispersions were formed after 50 min of illumination and exhibited an absorption peak at 550 nm, characteristic of plasmonic resonances in Au NPs. This is the first demonstration of CPL-induced photoreduction of metal ions in solution to synthesize chiral metal nanostructures. Meticulous studies are required to analyze the chirality of seemingly arbitrary shaped NPs forming racemic dispersions.

**Fig. 16:**
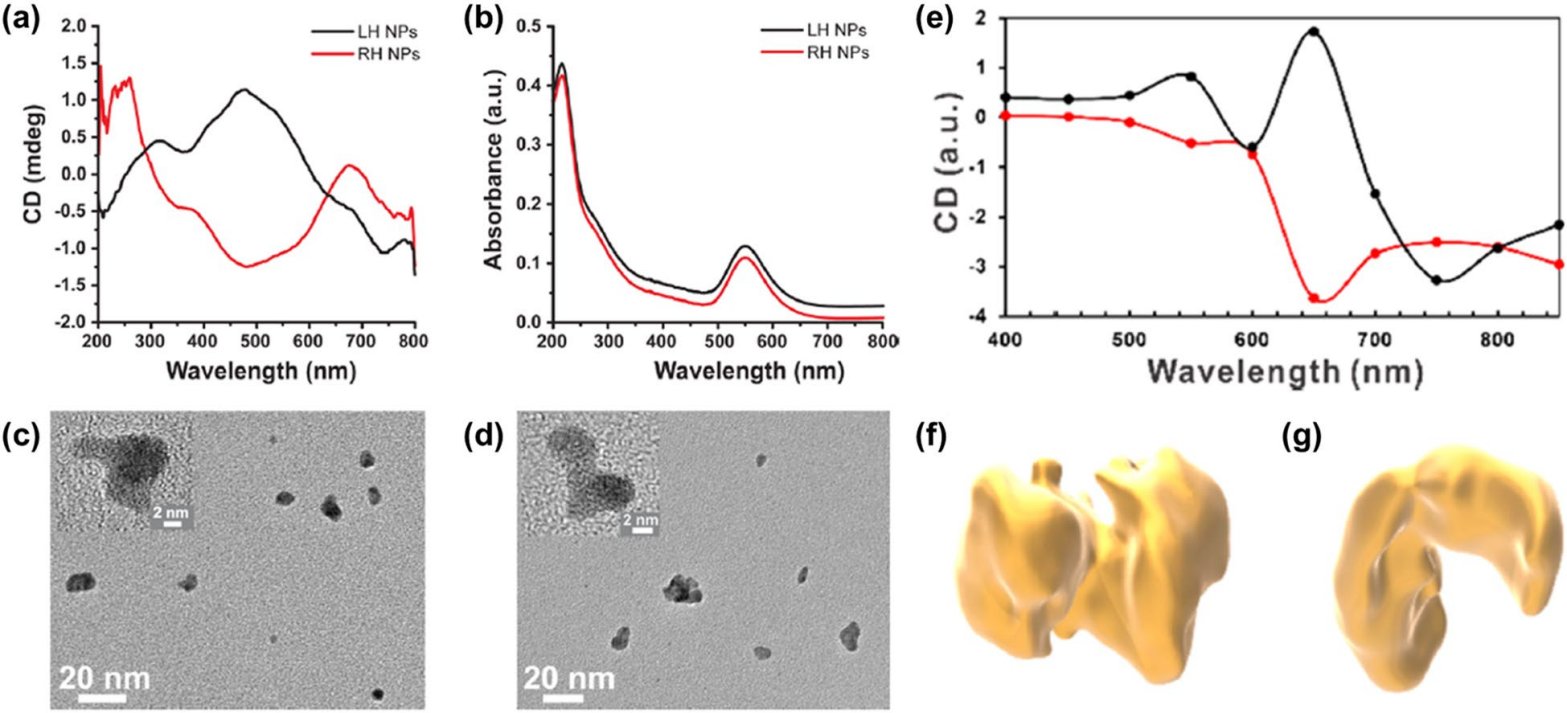
**a** CD spectra and **b** UV–vis absorbance spectra of dispersions formed under LCP (LH NP, black) and RCP (RH NP, red) (**c**, **d**) High resolution TEM images of NPs obtained with **c** LCP and **d** RCP illumination. **e** Calculated CD spectra with geometry of particle imported directly from tomographic reconstruction. **f**, **g** Experimentally obtained tomographic reconstructions of **f** LH and **g** RH gold nanostructures. Reproduced with permission from [18]. Copyright American Chemical Society, 2019

To investigate the mechanism of chirality transfer, TEM images and CD spectra of metallic NPs synthesized at various illumination times were studied [18]. (Fig. 16a–d) Au^3+^ was reduced to Au^0^ and ~ 2 nm NPs were formed after 5 min of illumination of (HAuCl_4_) solutions. After longer illumination, NPs grew in diameter (~ 3–5 nm) and coalesced into structures with complex shapes (~ 10–15 nm). The CD and UV–vis absorbance peak at 550 nm increased with the illumination time, and the CD bands disappeared after illumination with linearly polarized light. These results indicate the mechanism of chiral Au nanostructure formation. First, partial reduction of Au(III) by citrate to Au(0) clusters are photoactive at 543 nm. Au(0) clusters then grow to form ~ 3 nm Au NPs via photoinduced reduction. The resulting NPs acquired chiral geometrical bias due to circularly polarized photon energy, resulting in dynamic assemblies [95, 96].

Further examination was carried out using numerical solutions of the 3D vector Maxwell equations, based on the finite-element frequency-domain approach [18]. The integration of the Maxwell surface stress tensor suggests that CPL induces out-of-plane twisting forces exerted on NP assemblies to form chiral structures. Hence, polarization rotation of incident photons influences the direction of forces and, in turn, induces chiral assembly. The resulting assemblies with the chiral disposition of NPs maintain their shape by an NP–NP merger known as an oriented attachment and nonclassical crystallization processes*.* TEM tomography was also used to investigate the chirality transfer mechanism (Fig. 16f, g). The nano-assembly image shows complex geometries with non-obvious handedness. However, when tomography coordinates are directly imported into CD spectra calculations, the calculated spectra show a good correlation with the experimental results in terms of polarity and spectral placement of the CD peaks (Fig. 16e). Thus, for the light-induced metallic nanoparticle assembly, further optimization, such as light intensity and NP–NP interactions, can be investigated to prepare more uniformly shaped nanoscale assemblies [18]. These studies offer new methods for the colloidal synthesis of chiral structures, using light as a structural guide for semiconductor and metal NMs with the enantiomeric excess.

### Broad chiroptical activities from UV to SWIR

Chiral NMs can provide novel platforms for next-generation optical applications (i.e*.,* optical computing [97], telecommunication [98], bioimaging [99]) due to their high asymmetric interactions with CPL. In this perspective, tuning the wavelength of optical rotation maxima in the desired region is a key to achieving intended purposes. Chiroptical signals in the ultraviolet (UV) region primarily arise from the stereocenters of organic molecules on the surface of the inorganic core [100]. CD bands in the visible (Vis) region are observed in various chiral metal-, ceramic-, and semiconductor-based NMs because they typically absorb visible light as shown in previous sections. Recently, CD spectra in the infrared (IR) region (> 700 nm, especially near-infrared (NIR, 700–1700 nm) and shortwave-infrared (SWIR, 1700–2500 nm) windows) are reported by using building blocks of inorganic NMs with various geometries and dimensions at submicron-scale [101, 102]. In this section, we introduce two strategies of the bottom-up approach for synthesizing chiral inorganic NMs with the NIR-SWIR optical activity; (1) self-assembly and (2) templating approach.

**Fig. 17:**
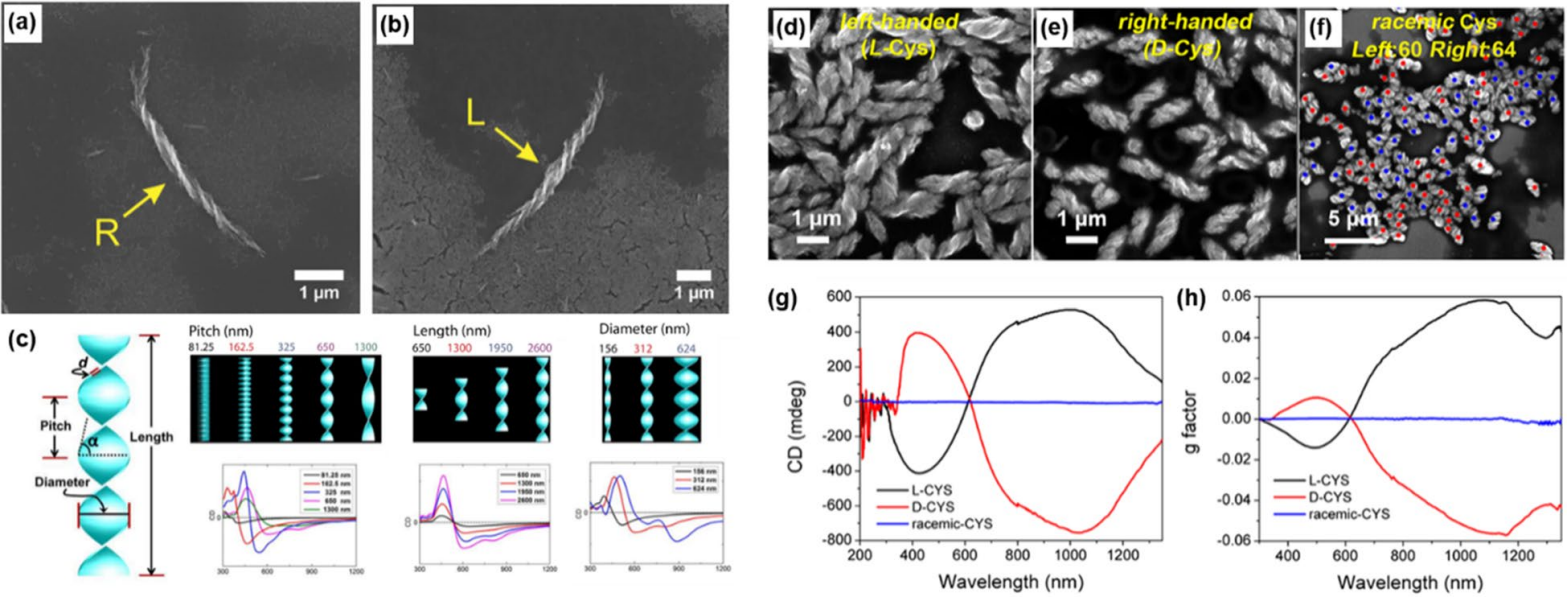
SEM images of **a** d-Cys CdTe NP assembly and **b** l-Cys CdTe NP assembly. **c** Illustration of the various geometrical parameters in a typical CdTe helix (**d** represents a thickness) and simulated CD spectra with geometrical parameters of the variables (pitch, length, and diameter). Reproduced with permission from [103]. Copyright 2017 AAAS. SEM images of helices made with** d** l-,** e** d-, and** f** *rac*-Cys surface ligands. For SEM images obtained in case of rac-Cys red and blue dots are used to mark the left-handed and right-handed helices, respectively. **g** CD and** h** g-factor spectra made with (black) l-, (red) d-, and (blue) rac-Cys surface ligands. Reproduced with permission from [104]. Copyright American Chemical Society, 2019

A self-assembly process, which is one of the practical methods of bottom-up synthesis, provides a versatile route to achieve chiral microstructures with various geometries from a broad set of inorganic NMs. The sub-micro dimensions of assembled structures are a suitable size to absorb IR light; thus, the self-assembly is a straightforward approach to synthesizing chiral inorganic NMs with optical activity in the IR region. Since the self-assembly process is sensitive to microenvironments such as temperature, pH, the concentration of reactants, the optical activity of assembled nanostructures is tunable. Kotov’s group synthesized chiral semiconductor CdS helices with an enantiomeric excess (ee) above 98% by assembling Cys-stabilized CdTe NPs in methanol (MeOH, Fig. 17a, b) [103]. The FDTD (finite-difference time-domain) simulation results demonstrated that the optical activity of the CdS helices depends on the structural factors such as pitch, length, diameter and thickness of helices (Fig. 17c). The same group reported a tunable optical activity of CdS helices with precise tailoring of such factors [104]. By modulating the assembly media (H_2_O:MeOH), the concentration of the ligand (Cys), coordination bridges (Cd^2+^ ion), and pH (6–11), the optimized left-, and right-handed CdS helices and their racemic mixtures were obtained with ee around 100% and CD spectra were tuned in the UV-NIR region (300–1300 nm) (Fig. 17d–f). The high asymmetric *g-*factor (~ 0.06) in the NIR region was achieved by optimizing the geometrical factors of the helices (Fig. 17g, h).

**Fig. 18:**
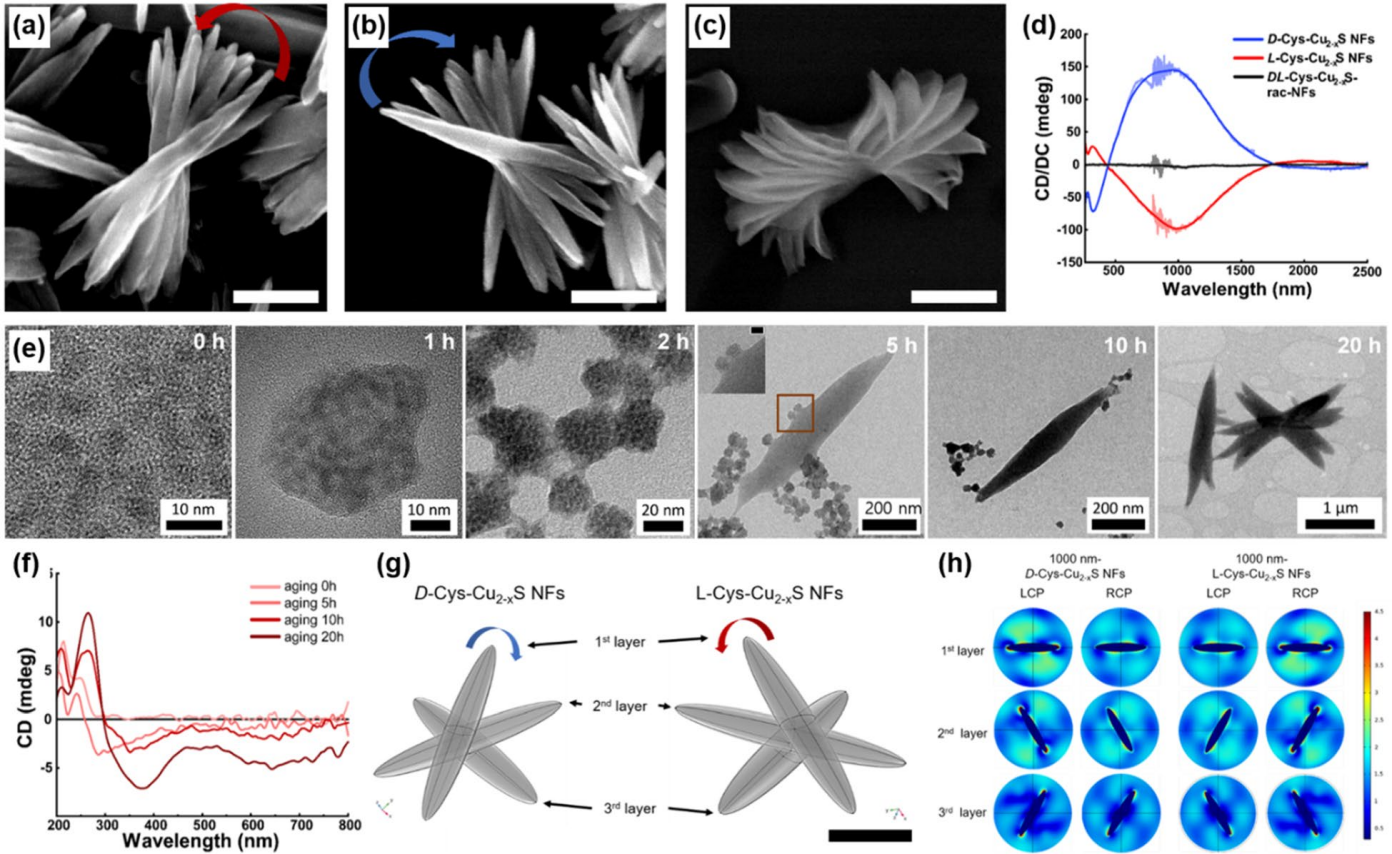
**a**–**c** SEM images of **a** l-Cys, **b** d-Cys, and **c** dl-Cys-Cu_2-x_S NFs. **d** CD spectra of thin films of NFs on quartz wafer (l-Cys (red), d-Cys (blue), dl-Cys (black)). Scale bars = 500 nm. **e** TEM images of the different stages during the NF formation. **f** CD spectra of the intermediate stages by aging time. **g** Top view of l-, d-Cys-Cu_2-x_S NFs models used in computational simulation (ellipsoid shaped nanoleaves stacked counterclockwise for l-Cys and clockwise d-Cys). Scale bars = 500 nm. **h** Electric field distribution of each layer in d-Cys-Cu_2-x_S NFs and l-Cys-Cu_2-x_S NFs model with 1000 nm LCP and RCP. Reproduced with permission from [105]. Copyright American Chemical Society, 2021

Yeom’s group reported the broader chiroptical activity (200–2500 nm, UV-SWIR region) by assembling l-, d-*,* and dl-Cys-Cu_2_S NPs into nanoflowers (NFs) in distilled water [105]. The resulting NFs are stacked in a counterclockwise (l-Cys), clockwise (d-Cys) direction or an achiral morphology (*rac*-Cys), and wide CD signals were observed due to their sub-microscale dimensions (Fig. 18a–d). The mechanism of chiroptical activity emergence was realized by TEM images and CD spectra of the structures of intermediate stages (Fig. 18e, f). In Fig. 17e, single l-Cys-Cu_2_S NPs form supraparticles (SPs) and then reorganize into nanoleaves (NLs) with intermediate products of SPs aggregates. With further aging, NLs stacked in a counterclockwise direction form NFs. Corresponding CD spectra of the intermediate products in Fig. 17f show that the original CD peaks of initial l-Cys-Cu_2_S NPs are remained, while new peaks arose during the self-assembly process. Such results demonstrate that the molecular chirality of Cys is transferred to microscale NFs step-by-step, serving NPs as the chiral building blocks. The chiroptical activity was originated from the structural chirality, which was validated by computational simulations of the differential absorptions of LCP and RCP in overall flower-like structures (Fig. 18g, h).

**Fig. 19:**
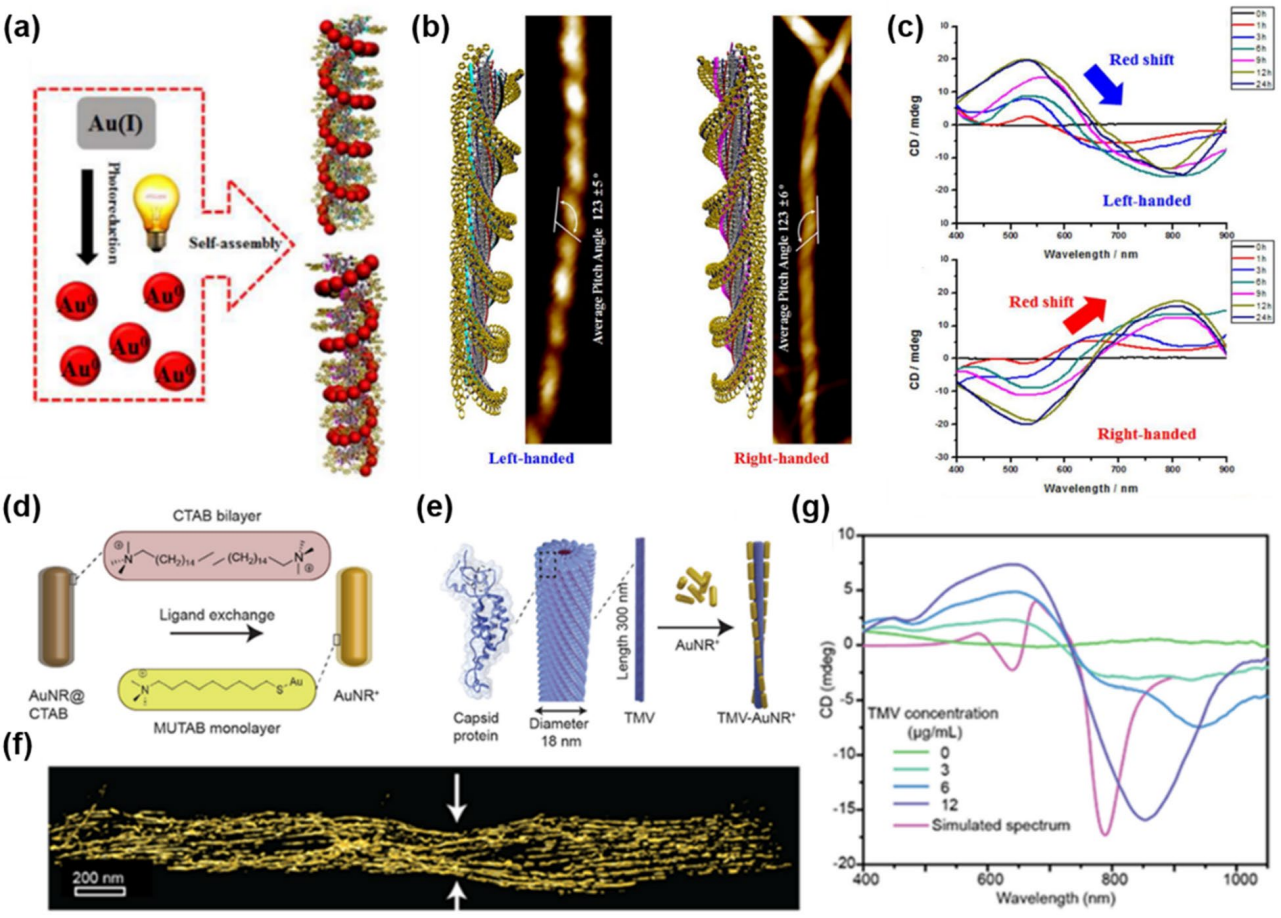
**a** Overview of helical nanofiber-guided chiral assembly of gold NPs. **b** Models (left) and AFM images (right) of helical nanofibers. **c** CD spectra of hydrogel containing Au (I) ions after UV irradiation for reductive growth of gold NPs on the helical nanofibers. Reproduced with permission from [14]. **d** Schematic of the functionalization of AuNR@CTAB with MUTAB via ligand exchange method. **e** Cartoon representation of the TMV structure and the schematic presentation of the electrostatic interaction between TMV and AuNR^+^. **f** 3D reconstruction image with arrows pointing to the helical twist. **g** Experimental CD spectra showing stronger signals with gradually higher TMV concentration, along with the simulated CD spectrum calculated from the AuNR^+^ coordinates obtained from **f**. Reproduced with permission from [15]

Using asymmetric nanostructures (i.e. supramolecules [106], DNA [107, 108], chiral mesoporous silica (CMS) [109]) as templates is another straightforward approach to synthesizing chiral NMs with IR chiroptical activity. Merging plasmonic properties of NMs into chirality of well-patterned structures of templates, a giant CD signal can be obtained and tuned. Jung’s group succeeded in assembling gold NPs in helical arrangement guided by supramolecular chirality of hydrogel (Fig. 19a) [110]. The chiral molecules of trimesoyltri(l-alanine) and trimesosoyltri(d-alanine) generated helicity in the nanofibers and gold ions were reduced on these templates by UV irradiation (Fig. 19b). The resulted helical array of gold NPs induces coupled plasmon absorption and thus CD spectra in the Vis–NIR region were obtained. Since the chiroptical activity of the system depends on plasmonic properties, CD signals were tuned by controlling the size of the golf NPs varying UV irradiation time, which tailored the wavelength of plasmon resonance absorption (Fig. 19c).

On the other hand, chiral template-assisted self-assembly of gold nanorods (AuNRs) into helical microwires was reported by Pradeep’s group by using Tobacco Mosaic Virus (TMV) particles with a negative-charged surface as chiral templates [111]. To assemble AuNRs along the TMV surface, AuNRs cationization was conducted by ligand exchange of cetyltrimethylammonium (CTAB) to (11-mercaptoundecyl)-*N*,*N*,*N*-trimethylammonium (MUTAB, Fig. 19d). In an ambient condition, TMV-AuNR^+^ composite was formed by the electrostatic attraction between anionic TMV and cationic AuNR^+^ (Fig. 19e). The control of the self-assembly by increasing stoichiometric ratios (TMV to AuNR^+^) showed that the increasing content of TMV led to gradual long-range helical microwires and CD spectra in the Vis–NIR region were observed due to the LSPRs of AuNRs (Fig. 19f, g).

IR photons have a lot of potentials in the biochemical field due to their high transparency to skins and biological tissues [112]. Taking advantages of their long penetration depth, NMs with photoluminescence in the NIR and SWIR region were utilized as biocompatible recording-patches [113] and nanothermometry [114], respectively. Thus, integrating chirality in NMs with IR chiroptical activity will pave the way for developing next-generation chiro-optical applications such as tumor evaluation [115], photothermal therapy [116], and neural stimulations [117]. Encryption using asymmetric interactions between NIR CPL and chiral NMs [118, 119] presented another potential of chiral NMs for bio-implantable data storage platforms.

### Chiroptical properties of mid- to far-infrared range

As the wavelength of light gets longer than near-IR, periodic chiral meta-atoms can be fabricated by a conventional top-down approach, i.e., lithography and deposition/etching process. Microelectromechanical (MEMS) devices have been considered a highly scaled and reproducible approach, especially in the fabrication of structures with sizes of a few micro- to macro scale. However, MEMS technique has intrinsic limitations in that it is hard to make complex 3D structures [120]. Since the optical activity of materials is originated from first-order spatial dispersion effect which is associated with the non-locality of the light–matter interaction [121], it is required to have sufficiently high 3D spatial variation in the micro structures for high optical rotational strength. Traditional MEMS devices are based on 2D patterning and deposition that needs to be transformed into 3D structures. There are recent developments of top-down approach using NMs for the mid- to far-infrared chirality and, in this section, we will revisit various strategies for 3D metamaterials from 2D patterns.

**Fig. 20:**
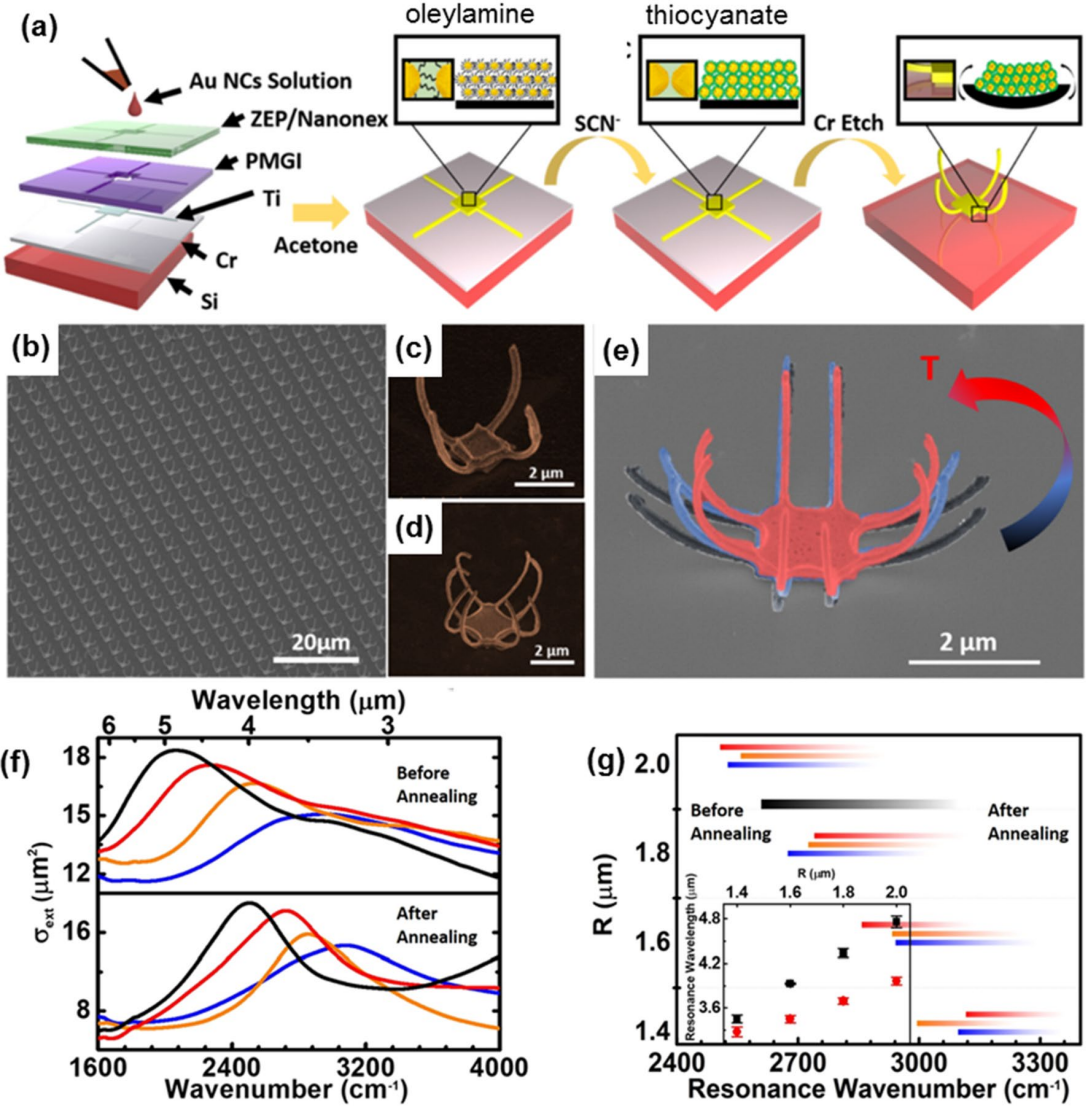
**a** Schematic of the fabrication of NP/metal bilayer metamaterials. **b**– **d** Representative SEM images of 3D metamaterials after. **e** Overlaid SEM images of meta-atoms; before annealing (black), annealing at 160 °C for 5 min (blue) and 60 min (red). **f** Extinction cross-sections for NP/metal metamaterials with varying R. Reproduced with permission from [120]. **g** Relation between resonance peak and the radius. The inset shows the shift of the resonance peak position. The black data points indicate samples before annealing and red, orange, and blue data points represent samples after annealing at 150, 120, and 90 °C for 24 h, respectively. Reproduced with permission from [122]

Kagan group [120, 122] investigated 3D metamaterials using NP assemblies. They used pre-patterned 2D planar structures in NP/metal bilayer structures as starting materials and triggered the folding of the bilayers by chemical and thermal treatments to reduce the spacing between NPs (Fig. 20). Representative SEM images of metamaterials showing tunability of plasmonic resonances are shown in Fig. 20b–e. Similar to conventional bimetallic strips, these micro-scale meta-atoms also can be mechanically deformed either by chemical and thermal methods. Chemically, they show that controlling the pre-strain in 2D NP/metal bilayer structures is possible by exchanging the length of ligands from longer (oleylamine) to shorter ones (thiocyanate), i.e., changing the volume of NP assembly. Also, heat treatment was exploited for the additional strain (Fig. 20e). Strong optical rotation was observed in the mid-IR range, and negative correlation between plasmonic resonance wavelength and the radius of curvature, R, was found (Fig. 20f, g). It is also confirmed by calculated spectra obtained from FDTD models that the curvature of the arm plays an indispensable role in the magnitudes of CD spectra and the transmission of LCP and RCP are determined by the induced polarity of electron currents in the curved metal layer.

Kirigami, the art of paper cut, has also recently arisen as a facile method of making 3D structures from pre-patterned 2D materials. Kirigami can be modulated in a totally mechanically elastic region and its reconfigurable capability with optical functionalities promise the untapped possibilities in optical metamaterials [123, 124]. Choi et. al. showed that that tunable optical elements fabricated from herringbone-patterned gold strips with kirigami cuts make the polarization modulation of terahertz (THz) radiation under the application of macroscale strain possible [124]. The THz region is a far-IR region and is often called a “THz gap” because the technology and optical materials for its emission and manipulation is infancy. A circularly polarized THz beam is indeed required for the measurement of the chiral vibration of molecules, but it has not been achieved due to the lack of chiral metamaterials [124–126].

The development of chiral NMs for mid- to far IR is actively being investigated because it holds importance from a number of perspectives, including from soft oscillation of bio-tissues, 5G telecommunication to cosmic rays for astronomy. Chiral modulators in this range could be used for secure high-bandwidth communication and non-invasive bio-imaging. Abundant molecular chirality in soft tissues is particularly suitable as a sample for gaining high contrast imaging when we use this range of circularly polarized light.

### Chiral phonon in NMs: from NIR to THz

When chiral NMs are interacting with relatively long wavelength of IR rays (from near-IR to THz waves), chiral phonons, phonons with intrinsic angular momenta, start to play an important role in light-matter interactions [126, 127]. Since the quantized energy levels of molecular vibrations are matched with a photon energy of IR light, the mirror-symmetric motion of chiral lattices interacting with linearly/circularly polarized infrared lights should be comprehended for fundamental physics and quantum photonic applications; however, little is known for chiral phonon-photon interactions. Phonon is one of the bosonic collective excitations and recently revealed that it can attain chirality [127]. Their existence has been theoretically proposed and studied in a number of 2D lattices [128], such as the Kekule lattice [129], the √3 × √3 hexagonal superlattice [130], and the Kagome lattice [131]. The phenomenon was also experimentally verified in atomically thin WSe_2_ through such as intervalley optical transitions of holes and single-photon emitter quantum dots [127, 132].

**Fig. 21:**
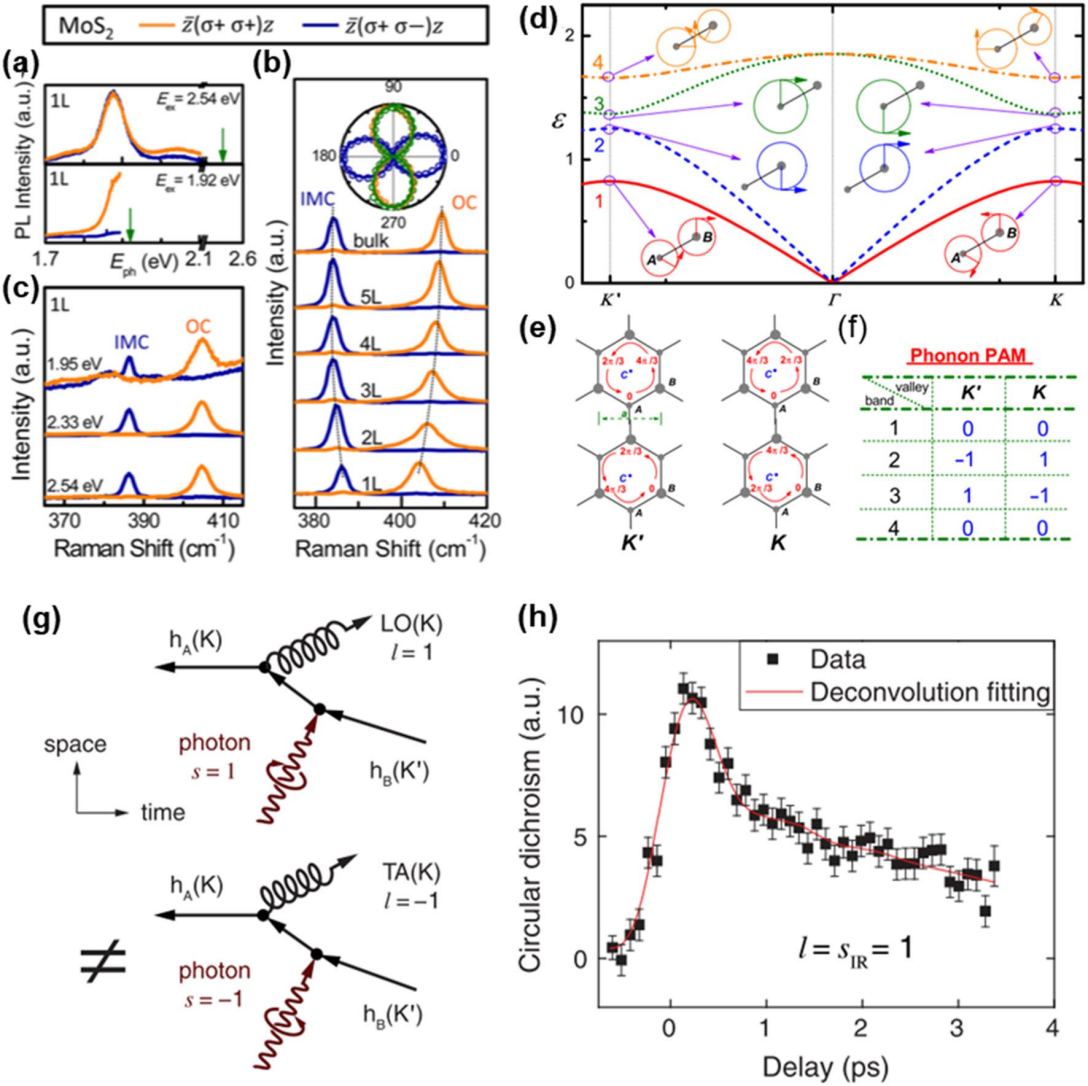
**a** The helicity-resolved photoluminescence of monolayer MoS_2_ at 80 K with excitation photon energy at 2.54 eV (upper) and 1.92 eV (lower). **b** The helicity-resolved Raman spectra (488 nm with σ + polarization) of MoS_2_ from monolayer to bulk. Inset: normalized angular dependence of the Rayleigh (green), IMC (blue), and OC phonon (orange) scattering intensities for monolayer MoS_2_. **c** Raman scattering with various excitation intensities. Reproduced with permission from [133]. **d** Phonon dispersion relation of a honeycomb AB lattice. The radii of circles denote vibration amplitudes; phase and rotation direction are included. **e** Phase correlation of the nonlocal phonon part for sublattice A (upper two panels) and sublattice B (lower two panels). Reproduced with permission from [128]. **f** PAM for bands 1 to 4 at valleys K’ and K. **g** Diagram of indirect optical transition for the LCP and RCP IR photons. **h** The measured CD in time-domain at 82 K. Reproduced with permission from [127]

The study on chiral phonons were initiated by the investigation of chirality reversal phenomenon of incident photons, which is observed in 2D semiconducting TMDs by helicity-resolved Raman scattering [128, 133]. Chen et al. [133] found that while the out-of-plane phonon (OC) with chalcogen atoms scattered photons has the same helicity as the incident photon, the in-plane phonon with metal and chalcogen atoms (IMC) shows opposite polarization rotation direction as shown in Fig. 21b. Moreover, this enantiomeric switching of incident photons is independent of layer number and incident photon energy (Fig. 21c), i.e., the IMC mode always drastically reverse the rotational direction of photons in contrast to the result of photoluminescence in Fig. 21a. This result implies that phonons involved in the intravalley scattering have chirality and the valley-photon helicity selection rule is robust depending on the symmetry of relevant phonon modes.

The first theoretical background of chiral phonons was proposed by Zhang, L. and Niu, Q. [128] from a 2D honeycomb lattice model, where each unit cell has two sublattices, A and B. They found circularly polarized sublattice vibrations at valleys (K and K’) which are mirror symmetric to each other as shown in Fig. 21d. Since the model chosen is graphene-like 2D lattice, any sublattice in a unit cell has a threefold rotation center (C_3_ symmetry) and thus, at a high-symmetry point, the pseudoangular momentum (PAM) must be $\pm 1$ or $0$, in other words, sublattices must create circularly polarized vibrations either left or right or they should be still (Fig. 21e and f).

Direct observation of chiral phonon mode was made by transient infrared (IR) spectroscopy in the monolayer WSe_2_ [127], which could measure polarization-selective absorption of IR in the time-domain (Fig. 21g and h). As expected, different IR absorption behavior of chiral phonons concerning the handedness of circular polarization of pump-probe was observed directly, indicating that the scattering cross sections for left and right CPLs are not identical. In addition, as revealed by helicity-resolved Raman scattering, it was confirmed that in-plane phonon mode or longitudinal optical (LO) phonons are dominant source of positive CD peak, especially, for delay time is longer than 0.8 ps in transient IR spectrum (Fig. 21h).

Chiral phonons are particularly intriguing not only are they strongly related to the collective motion of a number of atoms in NMs, but they are also associated with one of the actively pursued quantum information technologies, i.e., photon-phonon interaction. Chirality-dependent coupling between phonon and optical excitation is particularaly important since it can lead quantum control of collective excitations. An exemplary case is the quantum entanglement between chiral phonons of monolayer WSe_2_ and the single-photons from quantum dots which is induced by scattered phonons [132]. They found through the experiment of polarization-resolved PL spectroscopy that the scattered chiral phonons doubly degenerated (${l}_{photon}=\pm 1$) and they should be simultaneous projection of the polarization states of the photon, $\pm \sigma$ state as they are maximally entangled.

**Fig. 22:**
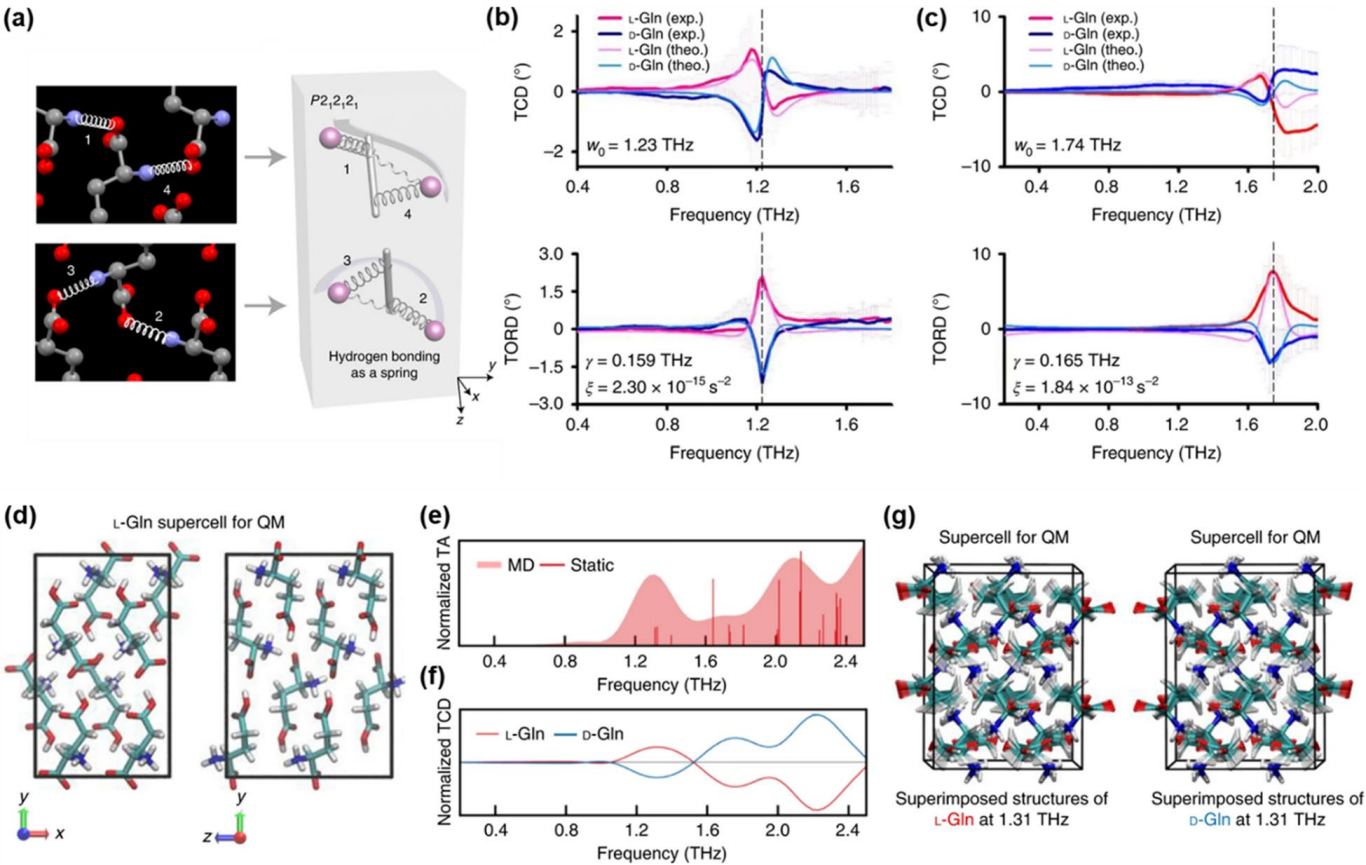
**a** Schematic view of hydrogen bonds in l-Glu crystal cell as an example chiral biocrystals. Hydrogen bonds represented as springs in the model used for a description of chiral phonons. **b**, **c** Experimental and calculated THz CD and THz ORD spectra of Glu (**b**) and Gln (**c**). **d** Supercell of l-Glu used for the quantum mechanical (QM) computations of the THz spectra. **e** Normalized THz absorption (TA) spectra obtained from the calculations of the l-Glu supercell in **d** using the normal-mode analysis (vertical lines) and molecular dynamics (MD simulations at the QM level (solid surfaces). **f** Normalized TCD spectra from the same MD simulations at the QM level used for TA in **e**. **g** Superimposed structural variations of the l-Glu molecules at 1.31 THz. Reproduced with permission from [126]

Chiral phonons can also be found in microcrystals and nanofibrils of biomolecules. Due to their large molecular weight segments or concerted motions of a large number of atoms, biomolecular complexes typically have their normal mode in the THz range [126]. With the recent development of THz circular dichroism (TCD), it enabled the observation of chiral phonon modes for microscale and nanoscale crystals of amino acids and peptides. Also, distinct bisignate peaks were found in biocrystals of such as glutamine (Gln) and glutamic acid (Glu) (Fig. 22a–c). The behavior of lattice vibrations in THz frequency can be visualized from atomistic computer simulations and their calculated spectra turned out to be well-matched with those of experimental results (Fig. 22d–g).

Phonons are universal in NMs with repeating units whether they are organic or inorganic. Phonons can strongly be correlated to electrons and photons in the system affecting numerous properties, such as electron–phonon interaction, electron transportation, Raman scattering, thermoelectric effect, superconductivity and even biological catalytic effect. Although the concept of chiral phonons is relatively new, but it has already played an important role in many physical, chemical systems as it was involved. Further investigation of chiral phonons will lead to the exploration and new development of enormous chiral applications.

## Conclusions and future outlook

Chiral NMs have become an important domain in nanotechnology with their exciting optical, electronic, and magnetic properties. For example, since the chiroptical properties of NMs can be governed by the versatile geometrical arrangements of NPs in 3D space [1], electronic and magnetic properties of the material composition, diverse types of chiral NMs, including semiconducting, metallic, ceramic, and magnetic NMs, have been broadly investigated. In particular, the long lifetime of the dipole moment of semiconducting NMs makes them possible to assemble into more complex chiral structures by chiral ligands or chiral light. Moreover, by illuminating chiral light during the synthesis process, the plasmonic state of gold NPs also allows them to be assembled into chiral structures with relatively irregular morphology because of their shorter lifetime compared to semiconducting NMs. Apart from the electronic properties of NMs, chiromagnetic NMs possess a greatly enhanced dissymmetric factor by the harmony of electronic and magnetic contributions to chirality.

However, despite these great achievements in developing various types of NMs with broad optical activity (from UV to THz), practical applications have not been actively achieved. Chiral NMs with reconfigurable and switchable electronic and magnetic properties by external stimuli are another unexplored research area for various applications; previously reported chiral NMs mostly showed the fixed electronic and magnetic properties [9, 14, 35]. In terms of scale, previous bottom-up assembly approaches with chiral NM building blocks are limited to submillimeter [12, 104, 111]. Once bulk scale assembly beyond micron with chiral NMs is realized, aside from nanometer-scale interactions with a biological system, studying the interactions between the assembled bulk structure using chiral NMs with biological tissues and their implantation applications will be an exciting field of research. Furthermore, understanding the chiroptical, electronic, and magnetic properties of complex NM structures is still challenging, due to the limitation in calculation capacity of density functional theory and ab initio [9, 11, 12, 134]. Thus, theoretical investigations of complex NM systems are demanded. These studies will open a new flourishing era of chiral NMs by providing a new degree of freedom in materials science evolution.

## Data Availability

Not applicable.
